# Tumor necrosis factor alpha mediates neuromuscular synapse elimination

**DOI:** 10.1038/s41421-020-0143-5

**Published:** 2020-03-03

**Authors:** Xiu-Qing Fu, Jian Peng, Ai-Hua Wang, Zhen-Ge Luo

**Affiliations:** 1 School of Life Science and Technology, ShanghaiTech University, Shanghai, 201210 China; 2 State Key Laboratory of Neuroscience, Center for Excellence in Brain Science and Intelligence Technology, Institute of Neuroscience, Chinese Academy of Sciences, Shanghai, 200031 China; 3 University of Chinese Academy of Sciences, Beijing, 100049 China

**Keywords:** Cell biology, Developmental biology

## Abstract

During the development of mammalian neuromuscular junction (NMJ), the original supernumerary axon inputs are gradually eliminated, finally leaving each muscle fiber innervated by a single axon terminal. However, the molecular cues that mediate the elimination of redundant axon inputs remain unclear. Here we show that tumor necrosis factor-α (TNFα) expressed in postsynaptic muscle cells plays an important role in presynaptic axonal elimination at the NMJ. We found that intramuscular injection of TNFα into the levator auris longus (LAL) muscles caused disassociation of presynaptic nerve terminals from the postsynaptic acetylcholine receptor (AChR) clusters. By contrast, genetic ablation of TNFα globally or specifically in skeletal muscle cells, but not in motoneurons or Schwann cells, delayed the synaptic elimination. Moreover, ablation of TNFα in muscle cells attenuated the tendency of activity-dependent competition in a motoneuron–muscle coculture system. These results suggest a role of postsynaptic TNFα in the elimination of redundant synaptic inputs.

## Introduction

Developmental synapse elimination during early postnatal life occurs widely in the central and peripheral nervous system, and is crucial for the formation of functional neural circuits^1–4^. This process is activity-dependent: inputs with relatively higher activity stabilized and inputs with lower activity gradually eliminated^5–7^.

The vertebrate neuromuscular junction (NMJ), a chemical synapse formed between the axon terminal of a motoneuron and a muscle fiber, has been a classical model in the study of synapse formation, elimination, and refinement^8–11^. At birth, each muscle fiber receives multiple innervations from spinal motoneurons. However, redundant inputs are gradually eliminated, leading to the singly innervated muscle cell within 2 weeks^12–15^. The competition among nerve terminals from different motoneurons is influenced by activity patterns and the relative efficacy of presynaptic inputs^5,6,16,17^. Recently, some retrograde factors, such as BDNF/proBDNF or Sama3A/Sama7A, expressed by postsynaptic cells are found to mediate presynaptic axonal elimination^15,18–22^. In addition, class I major histocompatibility complex (MHCI) has been reported to be involved in developmental synapse elimination at the NMJ^23,24^. Nevertheless, the molecular and cellular mechanisms regulating the competitions among nerve terminals on one single muscle fiber remain largely unknown.

Tumor necrosis factor-α (TNFα) is a pro-inflammatory cytokine acting as either a membrane-integrated ligand (mTNFα) or a soluble ligand (sTNFα) after cleavage of mTNFα by the metalloprotease TNF-α converting enzyme^25–27^. TNFα exerts biological functions via interaction with its cognate membrane receptor TNFα receptor type 1 (TNFR1) or TNFα receptor type 2 (TNFR2)^27–30^. mTNF is able to stimulate both receptors, whereas sTNF mainly acts on TNFR1 but not on TNFR2 despite high-affinity binding^30^. In the nervous system, TNFα is involved in various types of brain injury or neurodegeneration^31–33^. Moreover, previous studies have shown that glial TNFα regulates synaptic strength and mediates synaptic scaling by modulating transmitter release or postsynaptic receptor trafficking in cultured hippocampus neuron^34–37^. At the *Drosophila* NMJ, downregulation of TNF signaling attenuated NMJ degeneration mediated by disruption of neuronal skeleton protein spectrin/ankyrin^38^. Notably, several TNF members, including TNFα, LIGHT, and RANKL, inhibit neurite outgrowth and branching of cultured hippocampal neurons^39–41^. These studies indicate that TNFα is a possible candidate in neural refinement during early postnatal development. Thus, we try to understand the role of TNFα in developmental synapse elimination at the NMJ.

Here we show that TNFα expressed by postsynaptic muscle cells acts as a retrograde factor that induces presynaptic axonal elimination during the development of mouse neuromuscular synapses. Administration of TNFα into postnatal mouse levator auris longus (LAL) muscles caused separation of presynaptic nerve terminal from postsynaptic acetylcholine receptor (AChR) patches and decreased poly-innervated (PI) NMJs. However, genetic ablation of TNFα globally or specifically in muscle cells caused an opposite effect, leading to a significant delay of synapse elimination during the early postnatal 2 weeks. The role of TNFα was also determined in a motoneuron–muscle coculture system, in which activity-based synaptic competition was dampened by knockout of TNFα in muscle cells. Thus, TNFα plays an important role in synapse elimination during postnatal development.

## Results

### Activity-dependent expression of TNFα and its receptors in developing mouse NMJs

**Fig. 1:**
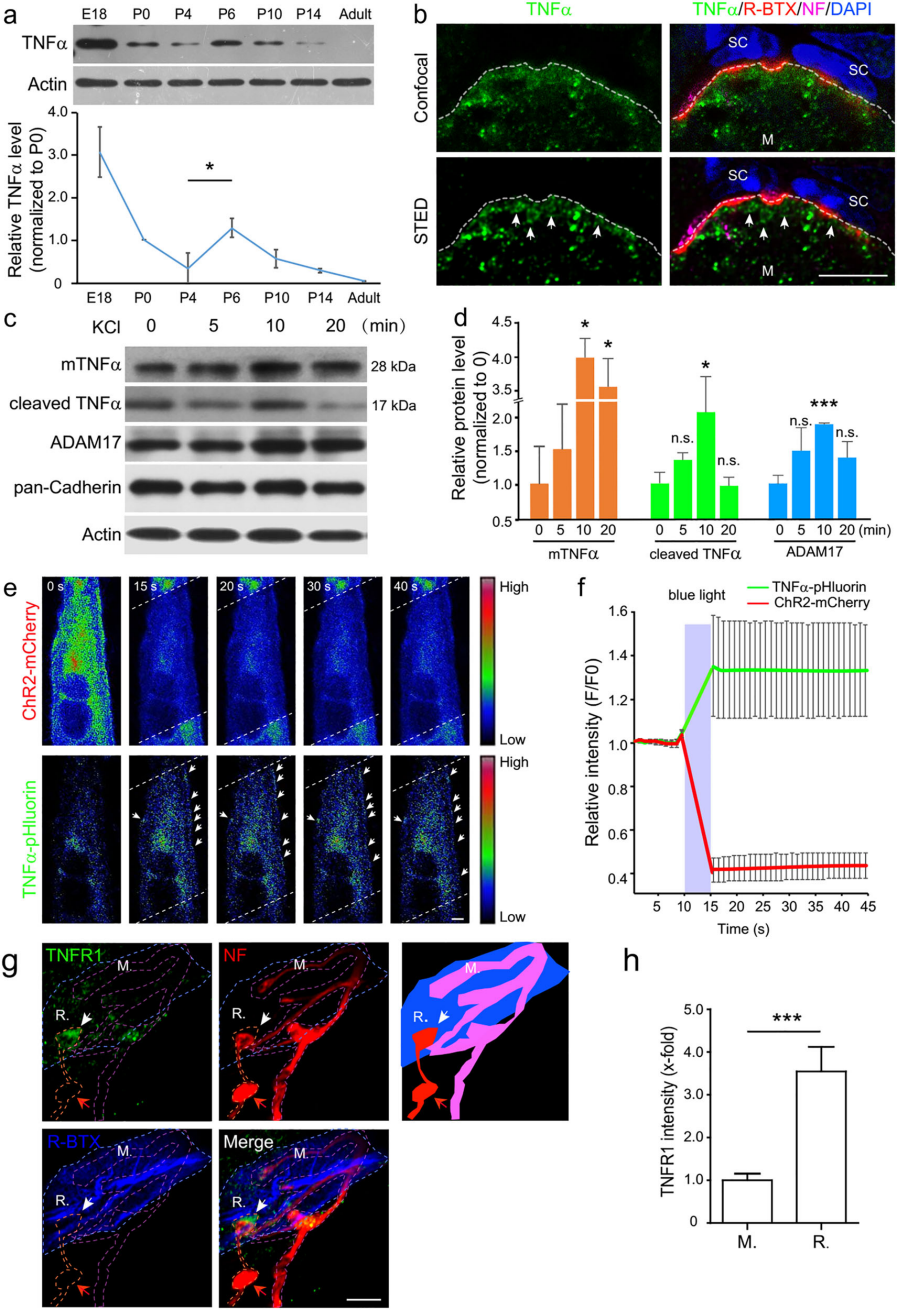
Expression of TNFα and its receptors in neuromuscular system. **a** Muscle homogenates (20 µg) of mice at indicated stages were subjected to immunoblotting (IB) with indicated antibodies. Relative level of TNFα protein normalized to P0 was quantified from three independent experiments. Data are means ± SEM. Mann–Whitney test was used to determine significance between P4 and P6. *P < 0.05. **b** Cross-sections of P6 sternocleidomastoid muscles were stained with R-BTX (red) and antibodies against TNFα (green) and neurofilament (NF, magenta). DAPI signals (blue) show the cell nuclei of terminal SCs. Top row: confocal images, bottom row: STED images. The notable vesicular distribution of TNFα was observed with STED method in postsynaptic muscle cells (white arrows). M muscle. White dash line: muscle membrane. Scale bar: 5 µm. **c**, **d** C2C12 myotubes were treated with 50 mM KCl for the indicated time. Then the membrane fractions of treated muscle cells were subjected to IB with antibodies against TNFα, ADAM17, pan-Cadherin, or Actin. Protein levels of mTNFα, cleaved TNFα, or ADAM17 relative to pan-Cadherin were quantified and compared with 0 min (**d**). Data are means ± SEM. Mann–Whitney test was used to determine significance. ***P *<* 0.001; *P *<* 0.05. **e**, **f** C2C12 myotubes co-transfected with TNFα-pHluorin and ChR2-mCherry were stimulated with ∼470 nm laser (white dash line area) to gate the ChR2 channel to activate muscle cells, followed by time-lapse imaging. Note the decrease in mCherry signals caused by photobleaching and increase in the fluorescence intensity of TNFα-pHluorin on the membrane of myotube (arrows) (**e**). Normalized fluorescence intensity of TNFα-pHluorin and ChR2-mCherry was quantified (**f**). Data presented are mean value of 25 cells with SEM. Stimulation period is marked with gray area on the graph. Scale bar: 10 µm. **g** The sternocleidomastoid muscles from P10 mice were stained with R-BTX (blue) and antibodies against TNFR1 (green) and NF (red). Note the expression of TNFR1 (white arrow) in the nerve terminal with retraction bulb (red arrow). The diagram outlines areas covered by retracting (R) and maintained terminal (M). Scale bar: 5 µm. **h** Relative intensity of TNFR1 relative to NF in retracting and maintained terminals was quantified. Data are shown as means ± SEM of 18 NMJs from 4 mice. Mann–Whitney test was used to determine significance. ***P *<* 0.001.

To investigate the role of TNFα in NMJ development, we first determined the expression patterns of TNFα in the mouse skeletal muscles and axons of motoneurons during the stage of presynaptic elimination. As shown in Fig. 1a, TNFα was highly expressed in soleus muscles of mice at embryonic day 18.5 (E18.5), declined after birth, bumped again at postnatal day 6 (P6), and then declined to undetectable level at the adult stage. Next, we performed immunohistochemistry for spatial localization of TNFα using confocal or stimulated emission depletion microscopy (STED) and found intense TNFα signals in muscle cells of P6 mice (Fig. 1b and Supplementary Fig. S1a). The intracellular TNFα puncta in muscle cells were reminiscent of vesicular TNFα signals during trafficking and secretion^42,43^ (Fig. 1b arrows and Supplementary Fig. S1a). Notably, TNFα signal was barely detected in terminal Schwann cells surrounding NMJs (Fig. 1b and Supplementary Fig. S1d, e). The specificity of TNFα and TNFR1 antibodies were confirmed by a negative signal in muscle samples from *TNFα*-knockout mice and in TNFR1-knockdown cells (Supplementary Fig. S1b-f). These data suggest that TNFα is mainly produced by postsynaptic muscle cells at NMJs.

Synaptic refinement during postnatal neuromuscular development is activity-dependent^5,6,16,17^. Therefore, we determined whether the production of TNFα by muscle cells is activity-dependent. To test this, differentiated C2C12 myotubes were stimulated with 50 mM KCl (high K^+^) for the indicated time (0~ 20 min), to induce depolarization. We found that the levels of both full-length (28 kDa) and cleaved TNFα (17 kDa) associated with the plasma membrane increased upon KCl stimulation (Fig. 1c). Furthermore, the protein level of ADAM17 endopeptidase, a main TNFα-processing enzyme that cleaves mTNFα into sTNFα, was also increased after KCl stimulation (Fig. 1c, d). This result suggests that the production and processing of TNFα might be activity-dependent. To further consolidate this conclusion, we measured the effect of light-gated cation channel Channelrhodopsin-2 (ChR2) on TNFα secretion from muscle cells. We found that upon pulsed blue light stimulation at a frequency mimicking physiological muscle firing for 5 s^5^, muscle cells expressing ChR2 tagged with mCherry exhibited a rise of calcium signals measured by GCaMP6f, which was used as a calcium probe^44^, and a bleach of mCherry signals (Supplementary Fig. S2a, b and Supplementary Movies S1 and 2). This result indicates that the light-gated ChR2 indeed increased activity in muscle cells (Supplementary Fig. S2a, b). Then, the same light-stimulation protocol was applied to muscle cells co-transfected with mCherry-ChR2 and TNFα tagged with pHluorin^45^, which has a relatively low fluorescence intensity in the acidic vesicle lumen and exhibits increased fluorescence signals when fused with the plasma membrane and exposed to the outer surface with higher pH. We analyzed the effects of activity on TNFα secretion, which was determined by measuring the dynamics of TNFα-pHluorin. We found that activated muscle cells exhibited a marked increase in TNFα-pHluorin signals, although mCherry signals were bleached (Fig. 1e, f and Supplementary Movies S3 and 4). Notably, the light-induced increase in TNFα-pHluorin signals was not observed in muscle cells co-expressing RFP (red fluorescent protein) and TNFα-pHluorin (Supplementary Fig. S2c, d), and light-gating of ChR2 did not increase the co-transfected YFP (yellow fluorescent protein) signals (Supplementary Fig. S2e, f). These results indicate the specificity of ChR2 action on TNFα-pHluorin. Next, to investigate whether TNFα expression is activity-dependent in muscle fibers of postnatal mice, we determined the effect of neuronal firing on TNFα production in postsynaptic muscle cells. For this purpose, pectoralis superficial muscles from P8 mice were transfected with plasmids encoding TNFα-pHluorin together with mCherry, to mark transfected cells using electroporation, followed by sequential electrical stimulation of innervating nerve fibers (Supplementary Fig. S3a–c). As shown in Supplementary Fig. S3d, pHluorin signals remained static prior to electrical stimulation. However, upon electrical stimulation of the nerves, innervated muscle cells exhibited subsequent increase in TNFα-pHluorin signals (Supplementary Fig. S3e–g). Taken together, enhanced activation of muscle cells promotes secretion of TNFα.

We also studied the expression pattern of TNFα receptors (TNFR1 and TNFR2) at NMJs of mice at P6 and found that TNFR1 was relatively highly expressed in nerve terminals with axon bulbs, which are hallmarks of retracting axons^6^, revealed by immunostaining (IS) with an antibody specifically recognizing TNFR1^46^ (Fig. 1g, h; Supplementary Fig. S1f, g and Supplementary Movie S5). In agreement with a recent report^47^, we also found that retracting axons exhibited microtubule disassembly as reflected from reduced levels of βIII-Tubulin (Supplementary Fig. S4a). Remarkable high TNFR1 signals were observed in the terminals of axons with low levels of βIII-Tubulin (Supplementary Fig. S4b, c and Supplementary Movie S6). Both TNFR1 and TNFR2 were localized in motor nerves in close opposition to the sites of postsynaptic AChRs (Supplementary Figs. S4d, S5a). Considering that competitive synapse elimination is activity-dependent, we determined the relationship between neuronal activity and levels of TNF receptors in cultured motoneurons. We found that both TNFR1 and TNFR2 were expressed in cultured motoneurons and, interestingly, treatment with high K^+^ (50 mM KCl, 30 min) to induce neuronal depolarization caused a decrease in the level of membrane-associated TNFR1 but an increase in TNFR2 (Supplementary Fig. S5b-d). Thus, the expression levels of TNFR1 and TNFR2 show distinct alterations related with neuronal activity.

### Administration of TNFα induces retraction of nerve terminals in LAL muscles

**Fig. 2:**
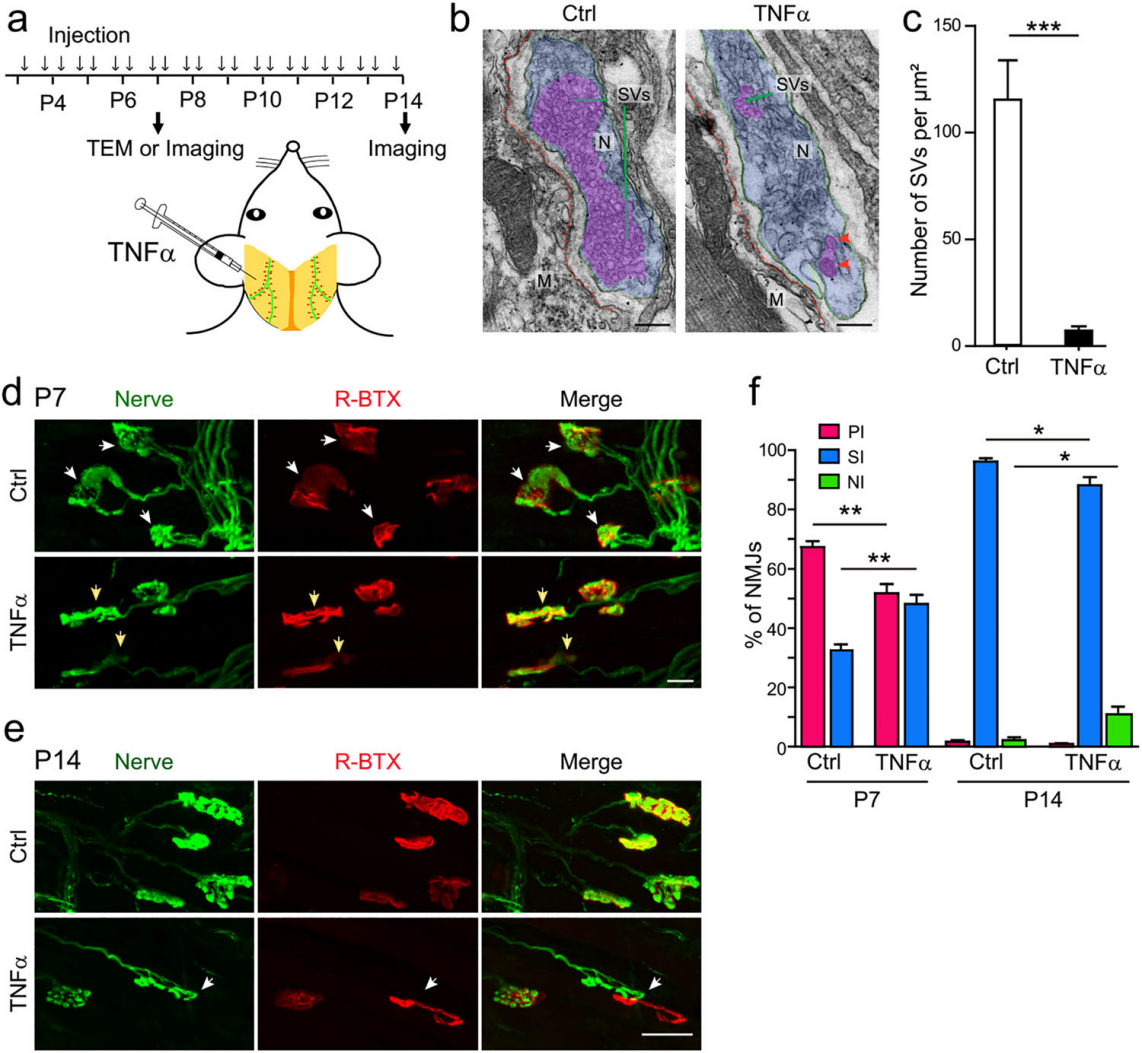
TNFα treatments promote synapse elimination at postnatal NMJs. **a** Schematic demonstration of experimental procedures. Left: LAL muscles of mice at P3 were injected with TNFα or bovine serum albumin (BSA) twice daily until P7 or P14, and then isolated for TEM or immunohistochemistry analysis. **b** EM images of NMJs of P7 mice after BSA (Ctrl) or TNFα treatment for 4 days. Blue area marks presynaptic nerve terminals with synaptic vesicles (SVs, carmine). Red dash lines delineate the postsynaptic membrane of myotubes. Note the detachment of nerve terminal from postsynaptic compartment and aberrant synaptic vesicles in TNFα-treated mice (red arrows). M: muscle, N: nerve terminal. Scale bar: 200 nm. **c** The number of synaptic vesicles in TNFα treatment group was markedly reduced compared with the BSA group. Data are shown as means ± SEM (16 NMJs from 4 mice for BSA group; 22 NMJs from 5 mice for TNFα group). Mann–Whitney test was used to determine significance. ***P *<* 0.001. **d**, **e** Drug-treated whole LAL muscles of mice at P7 (**d**) or P14 (**e**) were stained with R-BTX to label postsynaptic AChR (red) and antibodies against NF and synaptophysin to label nerve terminals (green). Note the poly-innervated NMJs (**d**, white arrows) in the control group and the single-innervated NMJs (**d**, yellow arrows), and the disassociation of axon terminals with AChR regions in TNFα-treated group (**e**, white arrows). Scale bar: 20 µm. **f** Quantification of synapse elimination at mouse NMJs following drug treatment. Data are presented as means ± SEM (P7: 644 NMJs from 6 mice of control group and 626 NMJs from 6 mice of TNFα group; P14: 676 NMJs from 4 mice of control group and 877 NMJs from 4 mice of TNFα group). Mann–Whitney test was used to determine significance. *P *<* 0.05, **P *<* 0.01. NI non-innervation, PI poly-innervation, SI single-innervation.

To determine the role of TNFα in postnatal synapse elimination at the NMJ, we took advantage of the LAL muscle, which is suitable for intramuscular application of exogenous factors or pharmacological agents via subcutaneous injection^48^. Interestingly, two recent studies using LAL muscles as a model to study synapse elimination through pharmacological manipulation have led to identification of a role of proBDNF in synapse elimination via TrkB/p75 neurotrophin receptors^21,49^. Given the negative phenotype of BDNF knockout mice in synapse elimination^21^, we hypothesized the presence of other retrograde factor, which is likely to be TNFα. To test this idea, purified TNFα protein was subcutaneously injected into the left LAL muscles from P3 to P13 twice daily (Fig. 2a). To examine the ultra-structure of NMJs, we did transmission electron microscopy (TEM) analysis for LAL muscles of P7 mice after treatment with TNFα or BSA (bovine serum albumin) for 4 days. As shown in Fig. 2b, LAL muscles were normally innervated by axon terminals with numerous synaptic vesicles in control mice. However, after TNFα treatment, the ultra-structure of axon terminals at the NMJ became abnormal with the lack of synaptic vesicles (Fig. 2b, c and Supplementary Fig. S6a). Next, we analyzed every single NMJ after drug administration for 4 days at P7. TNFα treatment caused a decrease of the percentage of NMJs that were innervated by ≥2 axons (PI) compared with the control group (Fig. 2d, f). At P14, almost all of the postsynaptic AChR patches were innervated by a single axon terminal (single-innervated, SI) in the BSA-injected control group and only residue postsynaptic sites remained non-innervated (Fig. 2e, f). Intriguingly, TNFα administration caused many postsynaptic AChR-enriched sites devoid of axon occupancy and a decrease of the percentage of SI NMJs (Fig. 2e, f and Supplementary Fig. S6b). These results indicate that TNFα can induce the retraction of axon terminals at the mouse NMJ.

### Genetic ablation of TNFα delays synapse elimination at mouse NMJs

To determine the necessity of TNFα in synapse elimination, we analyzed phenotypes of *TNFα* knockout (*TNFα*^*−/−*^) mice (Supplementary Fig. S7a, b). Similar to previous studies^50,51^, the homozygous *TNFα*^*−/*−^ mice were viable and fertile, showed no histological or morphological abnormality, and lived until adulthood. To investigate the function of TNFα in synapse formation, we analyzed the NMJs of mice at P0. We found that there was no difference in the number of AChR clusters between wild-type (WT) and *TNFα*-deficient mice in LAL muscles (Supplementary Fig. S7c-e), and the number of nerve arbors in diaphragm muscles (Supplementary Fig. S7f, g). Therefore, our data indicate that the absence of TNFα does not affect NMJ formation during an early stage of development.

**Fig. 3:**
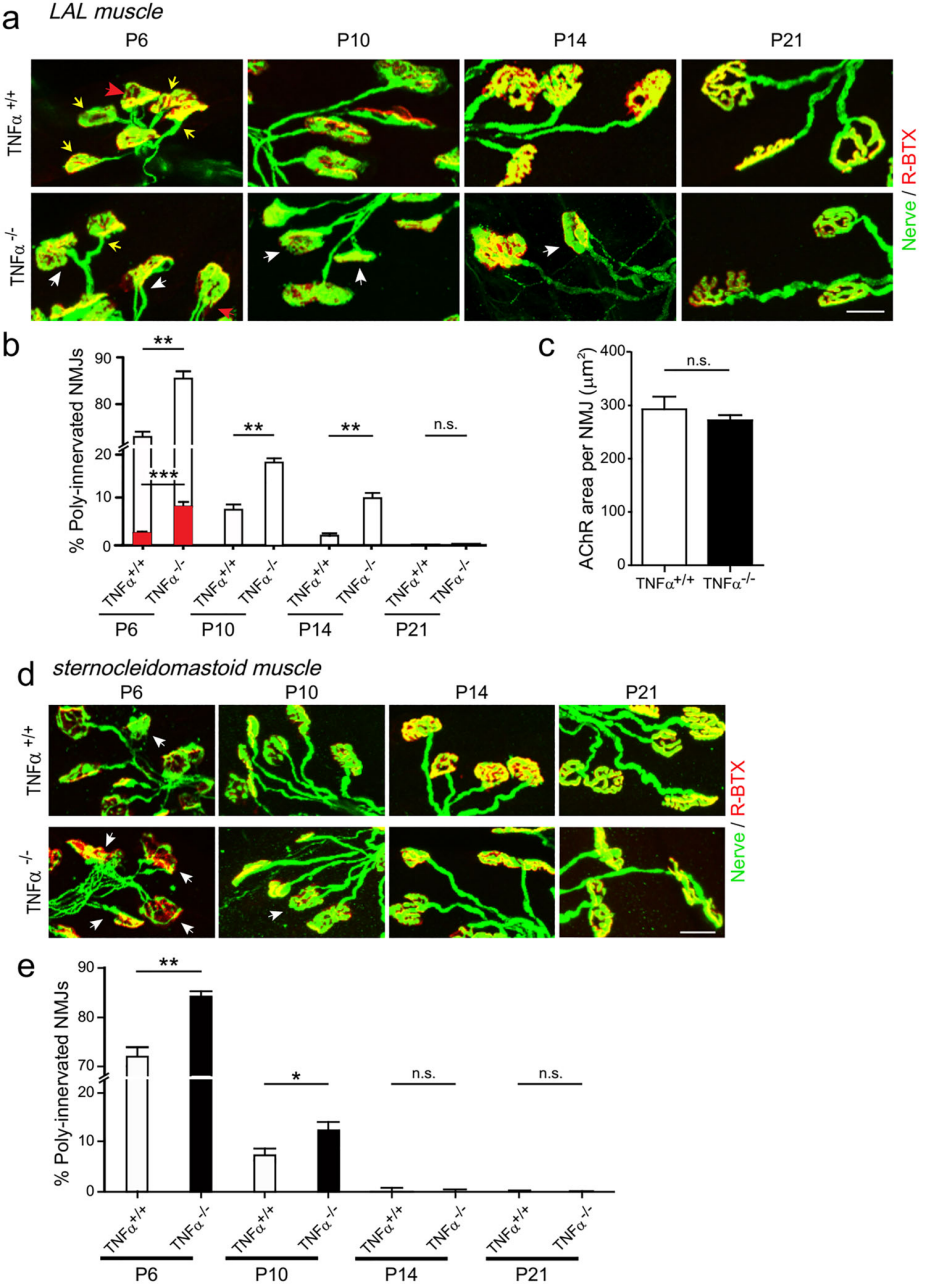
Synapse elimination is delayed in *TNFα*-knockout mice. **a** LAL muscles of wild-type (*TNFα*^*+/+*^) or *TNFα*-knockout (*TNFα*^*−/*−^) mice at indicated times (P6, P10, P14, and P21) were whole-mount stained with R-BTX (red) and antibodies against NF and Syn1 (Nerve, green). Arrows indicate the NMJs innervated by single axon (yellow), two axons (white), or more than two axons (red). Scale bar: 20 µm. **b** Quantification for the percentage of NMJs innervated by ≥2 axons at indicated postnatal days. Red bars represent percentage of NMJs innervated by >2 axons. Data are presented as means ± SEM (P6: 2079 NMJs from 4 *TNFα*^*+/+*^ mice, 2197 NMJs from 5 *TNFα*^*−/−*^ mice; P10: 1986 NMJs from 4 *TNFα*^*+/+*^ mice, 1906 NMJs from 3 *TNFα*^−*/−*^ mice; P14: 1760 NMJs from 5 *TNFα*^*+/+*^ mice, 1958 NMJs from 5 *TNFα*^*−/−*^ mice; P21: 2587 NMJs from 5 *TNFα*^*+/+*^ mice, 1897 NMJs from 3 *TNFα*^*−/−*^ mice). Mann–Whitney test was used to determine significance. **P *<* 0.01, ***P *<* 0.001, NS no significant difference. **c** Quantification for the average area of individual NMJ in P14 mice. Data are shown as means ± SEM from 91 NMJs in *TNFα*^*+/+*^ mice and 100 NMJs in *TNFα*^*−/−*^ mice. Mann–Whitney test was used to determine significance. NS no significant difference. **d** Sternocleidomastoid muscles of *TNFα*^*+/+*^ or *TNFα*^*−/−*^ mice at indicated time points were whole-mount stained with R-BTX (red) and antibodies against NF and Syn1 (Nerve, green). White arrows indicate the poly-innervated NMJs. Scale bar: 20 µm. **e** Quantification of the percentag**e** of poly-innervated NMJs. Data are shown as means ± SEM (P6: 3162 NMJs from 4 *TNFα*^*+/+*^ mice, 3514 NMJs from 6 *TNFα*^*−/−*^ mice; P10: 4416 NMJs from 8 *TNFα*^*+/+*^ mice, 3121 NMJs from 5 *TNFα*^*−/−*^ mice; P14: 3859 NMJs from 6 *TNFα*^*+/+*^ mice, 2204 NMJs from 3 *TNFα*^*−/−*^ mice; P21: 2532 NMJs from 5 *TNF*^*α+/+*^ mice, 1854 NMJs from 4 *TNFα*^*−/*−^ mice). Mann–Whitney test was used to determine significance. *P *<* 0.05, **P *<* 0.01, NS no significant difference.

Next, we examined synaptic patterns of NMJs at different postnatal stages. In line with previous observations^13,14^, most NMJs in the LAL muscle of WT neonatal mice were innervated by multiple axon terminals and the percentage of PI NMJs gradually decreased (Fig. 3a, b, d, e). At the end of postnatal week two, a vast majority of redundant axon inputs were gradually eliminated, leaving most NMJs SI and only minimal NMJs PI (Fig. 3a, b; 71.7 ± 0.3% at P6; 7.5 ± 1.5% at P10; 2.1 ± 0.5% at P14; 0.12 ± 0.04% at P21 in LAL muscles). Interestingly, the percentage of PI NMJs increased markedly in *TNFα*^−*/−*^ mice compared with WT mice littermates in the early two postnatal weeks (Fig. 3a, b; 85.5 ± 1.6% at P6; 18.2 ± 0.9% at P10; 10.3 ± 1.1% at P14; 0.3 ± 0.1% at P21 in LAL muscles, see arrowheads). Similarly, we also found the delay of synapse elimination in sternocleidomastoid muscles in *TNFα*^*−/−*^ mice (Fig. 3d, e). However, the area of individual NMJ marked by AChR showed no difference between WT and *TNFα*^*−/*−^ mice at P14 (Fig. 3c). These results suggest that TNFα plays an important role in presynaptic axon elimination during postnatal life at the NMJ.

### Muscle-derived TNFα is important for synapse elimination

**Fig. 4:**
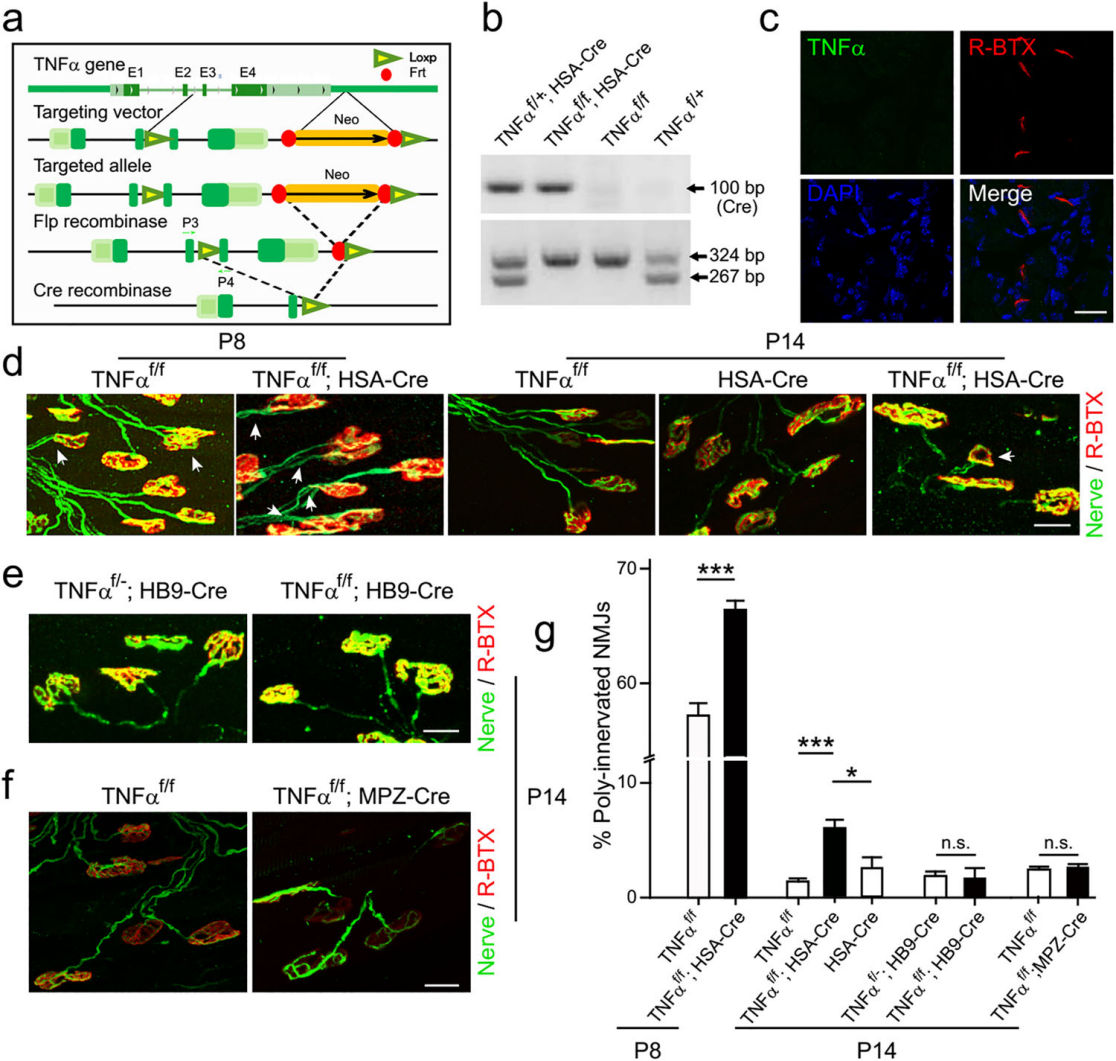
Genetic ablation of *TNFα* in skeletal muscle cells delays synapse elimination. **a** Strategy for gene targeting. A pair of LoxP sites flanked exon 3 and 4 of murine *TNFα* gene. The Cre-LoxP and Flp-Frt systems were used to ablate *TNFα* gene and selection marker Neo from the murine genome, respectively. P3 and P4 indicate sites corresponding to primers used for genotyping. **b** Genotyping of conditional knockout mice using PCR method. **c** Absence of TNFα expression in sternocleidomastoid muscles of *TNFα*^*f/f*^*; HSA-Cre* mice at P6. Scale bar: 20 µm. **d**–**f** LAL muscles of conditional knockout mice at indicated times (P8, P14) were whole-mount stained with R-BTX (red) and antibodies against NF and Syn1 (Nerve, green). Shown are example images of NMJs of mice with indicated genotypes at indicated times. White arrows indicate poly-innervated NMJs (**d**). Scale bars: 20 µm. **g** Quantification for the percentage of poly-innervated NMJs. Data are shown as means ± SEM (P8, 1877 NMJs from 4 *TNFα*^*f/f*^ mice, 2002 NMJs from 4 *TNFα*^*f/f*^*; HSA-Cre* mice; P14, 1036 NMJs from 6 *TNFα*^*f/f*^ mice, 3708 NMJs from 8 *TNFα*^*f/f*^*; HSA-Cre* mice, 1372 NMJs from 3 *HSA-Cre* mice, 1636 NMJs from 3 *TNFα*^*f/*−^*; HB9-Cre* mice, 636 NMJs from 3 *TNFα*^*f/f*^*; HB9-Cre* mice, 1707 NMJs from 3 *TNFα*^*f/f*^ mice, 3708 NMJs from 3 *TNFα*^*f/f*^*; MPZ-Cre* mice). Mann–Whitney test was used to determine significance. *P *<* 0.05, ***P *<* 0.001, NS no significant difference.

It has been hypothesized that a retrograde factor produced by postsynaptic muscle cells induced retractions of redundant axon terminals^15,18,20^. In addition, several studies indicated that terminal SCs participate in synapse elimination at the NMJ^52,53^. To identify the source of TNFα responsible for synapse elimination, we generated the *TNFα*^*f/f*^ mice with exon 3 and exon 4 of *TNFα* gene flanked by LoxP sites (Fig. 4a). Then, these mice were crossed with *HSA-Cre*, *MPZ-Cre*, or *HB9-Cre* mice to obtain mutant mice with specific ablation of *TNFα* in skeletal muscle cells, SCs, or motoneurons (Fig. 4b). Notably, TNF*α* signal was barely detectable in muscle samples from *TNFα*^*f/f*^*; HSA-Cre* mice (Fig. 4c), supporting that the expression of TNF*α* is in postsynaptic muscle cells again. Similar to global knockout mice, these conditional knockout mice had no obvious defects in synaptogenesis of NMJs at birth (data not shown). We analyzed the percentage of PI NMJs in LAL muscles at different stages and found that *TNFα*^*f/f*^*; HSA-Cre* mice exhibited a marked increase in PI NMJs compared with littermate controls at P8 and P14 (Fig. 4d, g). However, the absence of TNFα in SCs or motoneurons had no effect on synapse elimination (Fig. 4e–g). These results suggest that TNFα derived from muscle cells acts as a retrograde factor involved in postnatal synapse elimination at the NMJ.

### Role of TNFα signaling in activity-dependent synaptic competition in motoneuron–muscle coculture system

**Fig. 5:**
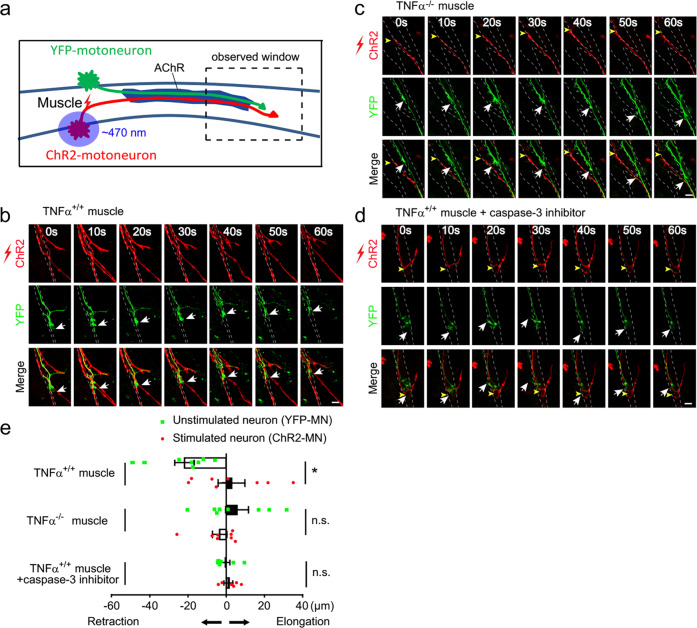
TNFα signaling is involved in activity-dependent competition in motoneuron–muscle coculture system. **a** Schematic demonstration of triplet set-up for optogenetic manipulation of cultured motoneurons. Pulsed blue light was applied to the soma of motoneurons expressing ChR2-mCherry to induce neuronal firing, followed by time-lapse imaging of distal regions of two axons co-innervating the same muscle cell. **b**, **c** Triplet cultures composed of ChR2-expressing motoneuron (ChR2-MN, red), YFP-expressing motoneuron (YFP-MN, green) and wild-type muscle cells (*TNFα*^*+/+*^) (**b**) or TNFα-deficient muscle cells (*TNFα*^*−/*−^) (**c**) were subjected to blue light stimulation. Time-lapse images were taken every 10 min after light stimulation. Note the retraction of YFP-MN terminal after light stimulation of ChR2-MN in triplet with *TNFα*^*+/+*^ muscles (white arrows in **b**) and elongation of both terminals contacted with *TNFα*^*−/−*^ muscle cells (arrows in **c**) during the observation period. Scale bar: 10 µm. **d** Triplet with *TNFα*^*+/+*^ muscles was treated with caspase-3 inhibitor DEVD (20 µM) during light stimulation and imaging. Scale bar: 10 µm. **e** Quantification for the distance of axonal retraction or elongation in 60 min after blue light stimulation of ChR2-MN in the triplets. Data are shown as means ± SEM of values from six to eight samples in each group. Mann–Whitney test was used to determine significance. *P *<* 0.05, NS no significant difference.

During the period of synapse elimination at the NMJ, multiple afferent nerves compete for the opportunity to form synapse with the single postsynaptic muscle cell and the nerve terminals with relative higher activity are deemed to be the favored competitor^6,7^. To determine whether TNFα is involved in the activity-dependent competition, we developed a triplet motoneuron–muscle coculture system, in which a single myotube was innervated by two motoneurons, which expressed ChR2-mCherry or YFP, respectively (Fig. 5a). After pulsed blue light stimulation, the behavior of innervating axons was observed using time-lapse microscope (Fig. 5b–d and Supplementary Fig. S8). We analyzed the situations with axons of both neurons intermingled on a muscle fiber, where they co-innervated an AChR patch labeled with low concentration of Rhodamine-labeled α-bungarotoxin (R-BTX) (Supplementary Fig. S8a). Remarkably, when the ChR2-expressing motoneuron (ChR2-MN) was activated by the blue light, the YFP axon innervating the same myotube gradually retracted (Fig. 5b, e; Supplementary Fig. S8a and Supplementary Movies S7, 8), usually with the appearance of retraction bulbs (Supplementary Fig. S8a and Supplementary Movie S8). However, this competition advantage did not occur in the cases where both axons co-innervating one muscle cell were either YFP or ChR2, without or with blue light stimulation (Supplementary Fig. S8b, c). These results are in agreement with the previous observation that more active inputs are favored competitors during synapse elimination^6^. Interestingly, this competitive advantage of ChR2-motoneurons was abrogated in the triplet cultures on muscle cells from *TNFα*^*−/*−^ mice (Fig. 5c, e and Supplementary Movie S9). Thus, muscle-derived TNFα participates in the competitive process of synapse elimination.

### Role of caspase-3 in synapse elimination at NMJs

**Fig. 6:**
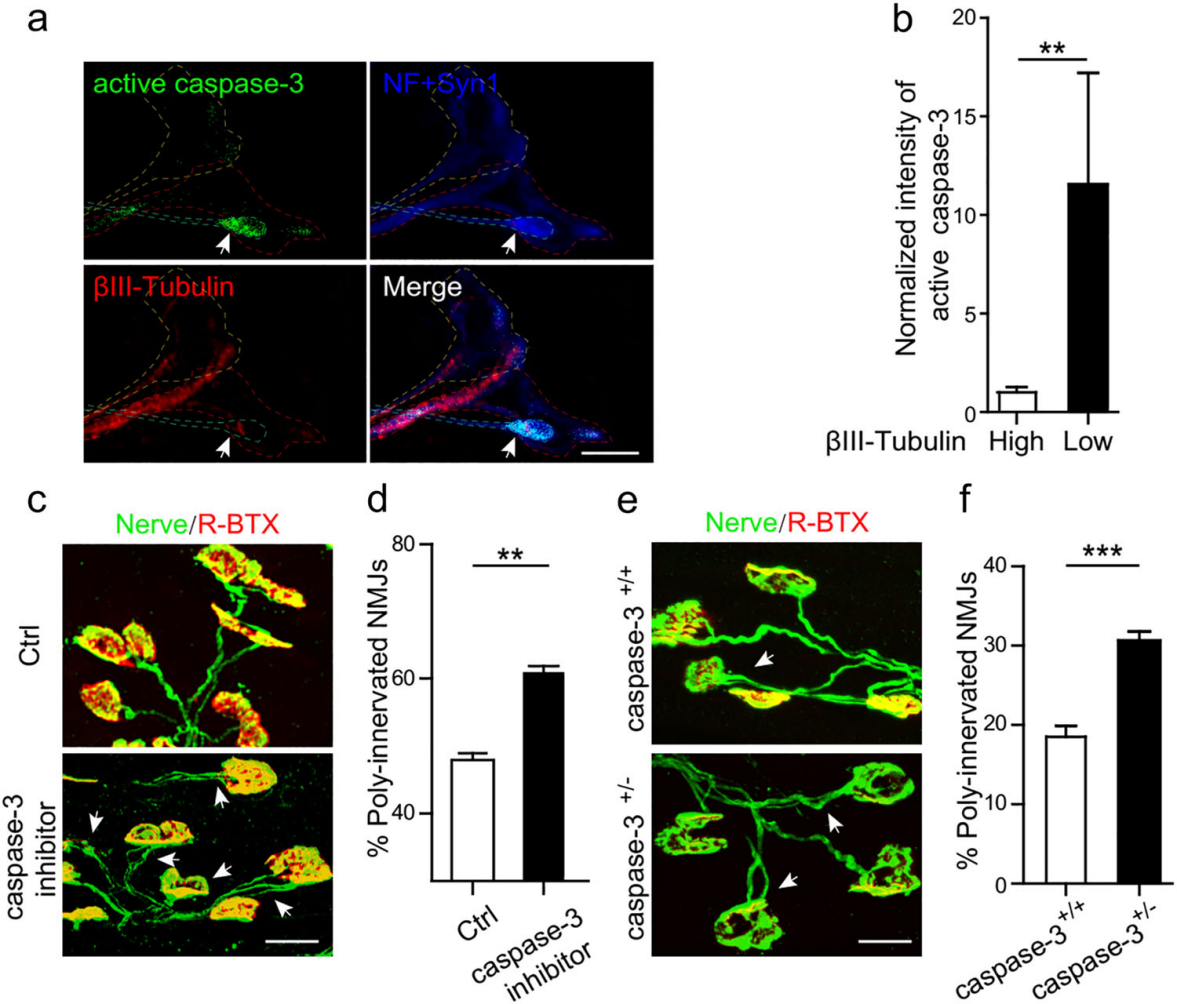
Caspase-3 is involved in NMJ synapse elimination. **a** The pectoralis superficial muscles from mice at P10 were incubated with FITC-DEVD-fmk for 1 h to probe active caspase-3 (green) and co-stained with indicated antibodies.Note the high level of active caspase-3 (arrows) in the retracting nerve terminal, which has lower level of βIII-Tubulin. Scale bar: 5 µm. **b** Quantification for normalized intensity of active caspase-3 relative to the terminal volume measured by NF and Syn1 signals. Data are shown as means ± SEM from ten NMJs in each group. Mann–Whitney test was used to determine significance. **P *<* 0.01. **c** Caspase-3 inhibitor DEVD (20 µM) or control vehicle DMSO was injected into LAL muscles twice daily from P3 and examined at P8 by staining with R-BTX (red) and antibodies against NF and Syn1 (Nerve, green). Note the poly-innervated NMJs (arrows) in representative images. Scale bar: 20 µm. **d** Quantification for the percentage of poly-innervated NMJs. Data are shown as means ± SEM (control: *n* = 5349 NMJs from 6 mice; caspase-3 inhibitor: *n* = 6132 NMJs from 7 mice). Mann–Whitney test was used to determine significance. **P *<* 0.01. **e** LAL muscles from wild**-**type or *caspase-3*^*+/*−^ mice at P9 were stained with R-BTX (red) and antibodies against NF and Syn1 (Nerve, green). White arrows indicate the poly-innervated NMJs. Scale bar: 20 µm. **f** Quantification for the percentage of poly-innervated NMJs. Data are shown as means ± SEM (*caspase-3*^*+/+*^: *n* = 4240 NMJs from 4 mice; *caspase-3*^*+/−*^: *n* = 4635 NMJs from 4 mice). Mann–Whitney test was used to determine significance. ***P *<* 0.001.

It is known that TNFα, through TNFR1 and mitochondria-independent pathway, activates caspase-8 and caspase-3 cascades to induce apoptosis^54–56^. To determine whether caspase-3 is also involved in the competitive process of presynaptic axon elimination, the triplets were incubated with caspase-3 inhibitor DEVD (20 µM) to block caspase-3 activity. Interestingly, the competitive advantage of ChR2-MN over YFP-MN was abrogated in DEVD-treated samples (Fig. 5d, e and Supplementary Movie S10). We also found that the active caspase-3 was mainly localized in the terminals of retracting axons with low level of βIII-Tubulin (Fig. 6a, b). These data indicated that caspase-3 is involved in synapse elimination. To investigate the role of caspase-3 in vivo, we subcutaneously injected DEVD (20 µM) into LAL muscles from P3 twice daily until P8. We found that the percentage of PI NMJs was markedly increased in DEVD-treated mice compared with control mice (Fig. 6c, d). Furthermore, *caspase-3*^*+/−*^ mice exhibited an increase in the percentage of PI NMJs compared with WT littermates at P9 (Fig. 6e, f). This effect was not due to the blockade on developmental motoneuron loss, because nerve branches were similar between WT and *caspase-3*^*+/*−^ mice (Supplementary Fig. S7h, i). These results suggest that TNFα/caspase-3 signaling is involved in postnatal synapse elimination during NMJ development.

## Discussion

**Fig. 7:**
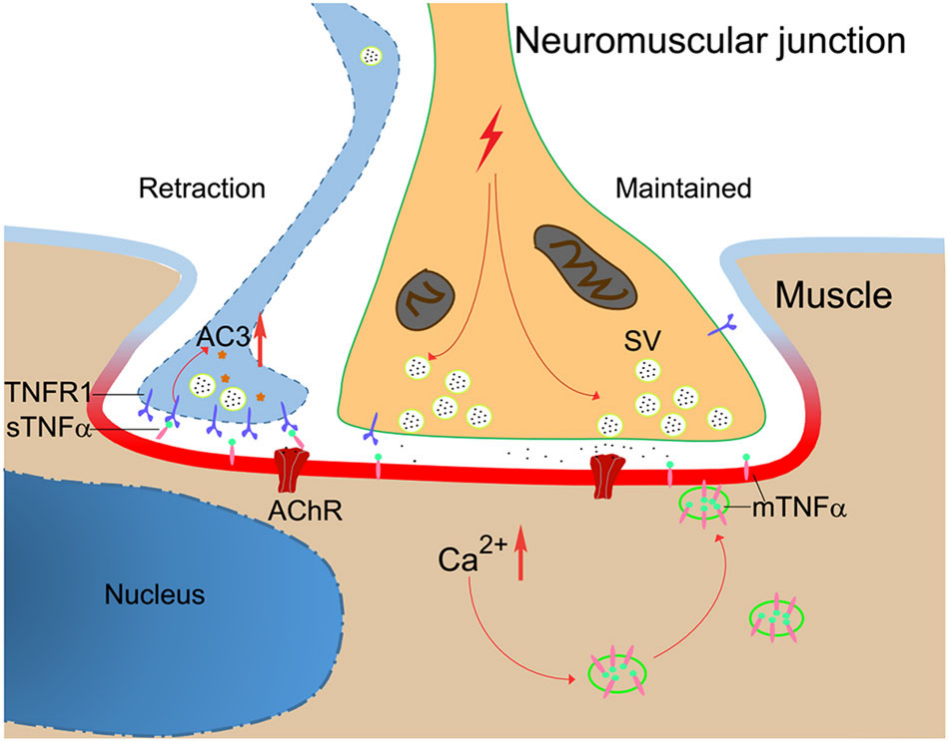
Proposed model of TNFα action in synapse elimination at the NMJ. Synaptic activity induces TNFα expression from postsynaptic muscle cells, which in turn promotes pruning of nerve terminals with relatively low activity, high expression of TNFR1, and the activation of caspase-3. AC3, active caspase-3.

Synapse elimination is an important process for maturation and refinement of neural circuits during the development of the nervous system^2,8^. In rodents, more than two axon terminals compete for the same postsynaptic muscle fiber but most of them are destined for the elimination only leaving the muscle fiber mono-innervated^14,57^. The molecular mechanism that regulates this synapse elimination remains poorly understood. The results presented in this work suggest a role of inflammatory factor TNFα in mediating developmental synapse elimination at the NMJ (see Fig. 7 for the model). This conclusion is supported by several lines of evidences: first, the expression patterns of TNFα in skeletal muscles coincided with the period of inputs pruning; second, the expression of TNFα in muscle cells and receptors in motoneurons are activity-dependent; third, ectopic TNFα injection induced removal of presynaptic terminals; finally, and most importantly, genetic ablation of TNFα, in particular in the muscle cells, postponed the postnatal elimination of neuromuscular synapses.

### TNFα as a retrograde factor mediating presynaptic elimination during NMJ development

Synapse elimination at NMJs or axonal pruning in retino-geniculate refinement during early postnatal development have been suggested to be mediated by retrograde factors produced by postsynaptic cell^15,20,21,58^. It has been hypothesized that synapse elimination or axonal pruning at NMJs during early postnatal development may be mediated by a “synaptotoxin” produced by postsynaptic cell to remove presynaptic terminals or the competition for limited amount of a “synaptotrophin” to stabilize axon terminals^15^. Although recent studies suggested that proBDNF might be the punishment signal and the mature BDNF might be the “synaptotrophin” signal, the genetic evidence supporting this conclusion is still lacking^21,58^. Our study has provided ample evidence supporting that TNFα derived from postsynaptic muscle cell regulates synapse elimination as a synaptotoxin at the NMJ. It is known that TNFα is a cytokine that plays an important role in host defense, inflammation, and immune balance^59^. TNFα participates in a variety of trauma or diseases caused by acute or chronic inflammation, including neurodegenerative diseases, such as Alzheimer’s disease, Parkinson’s disease, amyotrophic lateral sclerosis^57^, and multiple sclerosis^31,33^. It is also involved in synaptic plasticity and regeneration in the nervous system^34,36,38^. In *Drosophila*, TNFα promotes axon collapse through mitochondria-caspase signaling pathway under cellular stress conditions^38^. Here we show that TNFα acts as a retrograde factor regulating developmental synapse elimination at the NMJ. During the period of postnatal synapse elimination, TNFα is mainly produced by postsynaptic muscle cells. Considering that the developmental NMJ elimination is activity-dependent^5,6,16,17^, the regulation of TNFα expression by activity of muscle cells (Fig. 1c–f) makes it an ideal candidate as a “punishment” factor. In line with this hypothesis, genetic ablation of *TNFα* gene in muscle cells, but not in SCs or motoneurons, postponed the postnatal synapse elimination (Fig. 4). Although we failed to observe the presence and the role of TNFα in terminal SCs, this study does not exclude the involvement of SCs in synapse elimination through the expression of other factors as reported in previous studies^52,53,60^. Alternatively, it remains possible that TNFα mediates presynaptic elimination through SCs that may also express TNF receptors.

### Mechanism of TNFα presynaptic elimination during NMJ development

How does the selective elimination of inappropriate synaptic connections happen? Interestingly, several classes of immune molecules, including C1q, the initiating protein in the classical complement cascade, and MHCI, play important roles in developmental synapse elimination^61–64^. Although synapse elimination at the NMJ occurred normally in mice deficient for pivotal protein of the complement cascade C3^61^, MHCI appeared to be involved^23^. In addition, glutamatergic transmission via *N*-methyl-d-aspartate (NMDA) receptors seemed to be involved in the removal of excess innervation at the end plate^65^. It would be of interest to determine the interplay between NMDA and TNFα signaling, e.g., whether NMDA-induced Ca^2+^ influx regulates TNFα expression or processing from postsynaptic muscle cells, and whether TNFα regulates membrane localization of glutamate receptors. Indeed, it has been shown that TNFα participates in synaptic scaling via upregulating the surface level of AMPAR^36^.

How does TNFα select which inputs to be “punished”? TNFα signal is transduced via two distinct receptors, TNFR1 and TNFR2, which mediate different downstream signaling pathways and control the life and death balance of cells^29,66^. It is known that TNFR1, through the cytoplasmic domain, recruits several adaptor protein, including TNFR1-associated death domain protein, receptor-interacting protein 1, and TNF-receptor-associated factor 2, and activates caspase-8 and caspase-3 cascades to mediate mitochondria-independent apoptosis^54–56^. Of note, several studies have identified non-apoptotic roles of caspase-3 in the elimination of postsynaptic structures^67,68^. The relatively high expression of TNFR1 and active caspase-3 in retracting axons (Figs. 1g and 6a), and reverse correlation between the expression level of TNFR1 with neuronal activity (Supplementary Fig. S5b, c) suggest that different state of TNFR1/caspase-3 signaling may determine the competition outcome. In line with this notion, inhibition of caspase-3 interfered with synapse elimination in triplet culture system and in vivo (Figs. 5d, e and 6c-f). In contrast to TNFR1, the expression of TNFR2 in motoneurons was enhanced by neuronal activity (Supplementary Fig. S5b, c). It remains to be investigated whether the differential expression patterns of TNF receptors, different responses to membrane-bound or soluble form of TNFα ligand, and complex downstream signaling network discriminate initial axonal inputs encoding for maintenance or retraction. It has been shown that branch-specific disassembly of axonal microtubule is involved in developmental synapse elimination at the NMJ^47^. It would be of interest to establish the link between TNFα signaling and axonal microtubule stability.

In conclusion, our study uncovers an important role of TNFα in regulating synapse elimination at NMJs. Nevertheless, this study does not exclude the involvement of other factors expressed in muscle cells, SCs, or neurons in synapse elimination. In addition to the reported role in regulating synaptic scaling^36^, TNFα may also participate in structural synaptic plasticity in the central nervous system, such as the retino-geniculate refinement^34,69^.

## Materials and methods

### Mice

Animal experiments were conducted according to the guidelines of Animal Use and Care Committees of Institute of Neuroscience, Chinese Academy of Sciences. *TNFα*-knockout mice (JAX003008), *MPZ-Cre* transgenic mice (JAX017928), *HB9-Cre* transgenic mice (JAX00600), and *caspase-3*-knockout mice (JAX 006233) were from Jackson Laboratory. The *HSA-Cre* mice were introduced in previous studies^70–72^. The *TNF*^*floxed/floxed*^ (*TNF*^*f/f*^) mice with loxP sites flanking exons 3 and 4 of *TNFα* gene were generated by Biocytogen Company (Beijing, China). The following primers were used for the genotyping of conditional knockout mice: *Cre*, 5′-GCGGTCTGGCAGTAAAAACTATC-3′ (P1) and 5′-GTGAAACAGCATTGCTGTCACTT-3′ (P2); *TNFα*, 5′-CTACACAGAAGTTCCCAAATGGC-3′ (P3), and 5′-GTCACTCGAATTTTGAGAAGATGATCC-3′ (P4). All mice analyzed were in the *C57BL/6J* background.

### Reagents and antibodies

Antibodies were from the following: Abcam (TNFα-ab1793 for western blotting, WB; TNFR2-ab7369 for WB and immunostaining, IS), Novus (TNFα-NBP1-19532 for IS), Cell Signaling (Neurofilament-L-NF-2837s for IS, Synapsin-1-(Syn1)-5297s for IS), Invitrogen (Synaptophysin-(SYP)-18-0130 for IS), Chemicon (Actin-MAB1501 for WB), Kangcheng Biotechnology (GAPDH-kc-5G4 for WB), and Sigma (pan-Cadherin-C1821 for WB). DAPI (4′,6-diamidino-2-phenylindole) was from Beyotime. The secondary antibodies used in immunostaining were from Invitrogen. Horserdish peroxidase-conjugated secondary antibodies were from Millipore. Recombinant agrin and goat-anti-TNFR1 (AF-425-PB for IS) were from R&D. R-BTX was from Invitrogen. Caspase-3 inhibitor Ac-DEVD-cmk (shorted as DEVD, 218750) and caspase-3 activity detection probe (FITC-DEVD-FMK, JA5700) were from Merk/Calbiochen. pTNFα-pHluorin, pCAGGS-RFP, and pCAGGS-eYFP were constructed in this work. pCAGGS-ChR2-mCherry and pCAGGS-mCherry were gifts from Dr Zilong Qiu. pGP-CMV-GCaMP6f was from GENIE Project (Addgene plasmid #40755).

### Total and membrane protein extraction

The limb muscle of mice were homogenized in cold lysis buffer containing 50 mM Tris-HCl, pH 7.5, 150 mM NaCl, 1% Nonidet P-40, 0.5% sodium deoxycholate, and protease inhibitors Cocktail set III (539134, Merk/Millipore). Membrane proteins of C2C12 myotubes or cultured primary motoneurons were prepared by using plasma membrane protein extraction kit (k268-50, Biovision) and subjected to immunoblotting experiments using indicated antibodies.

### Muscle cell culture, transfection, and optogenetic manipulation

C2C12 muscle cells or primary muscle cells from P0 mice were cultured in Dulbecco’s modified Eagle’s medium (DMEM) containing 20% fetal bovine serum and induced for differentiation in DMEM medium containing 3% horse serum. C2C12 myoblasts were transfected with plasmids (TNFα-pHluorin plus ChR2-mCherry, RFP plus TNFα-pHluorin, YFP plus ChR2-mCherry, or ChR2-mCherry plus GCaMP6f) using Lipofectamine® 2000 (11668-019, ThermoFisher), followed by differentiation into myotubes and live-imaging analysis under Nikon FN1 laser scanning confocal microscope (NIR Apo 40x DIC Water N.A. 0.8). For triple-color imaging, excitation laser of 488 nm (Emission spectrum-Em: 500–550 nm), 543 nm (Em: 570–620 nm), and 640 nm (Em: 663–738 nm) were used. ChR2-expressed myotube were photo-activated with ∼ 470 nm laser (2 Hz, 5 ms per pulse, 10 pulses, 60 s interval), followed by imaging analysis for fluorescence dynamics of GCaMP6f or TNFα-pHluorin (excitation 488 nm; EM: 500–550 nm) by recording every picture per second. Post-acquisition images were processed with Rainbow RGB of Fiji software to obtain the pseudo-color images.

### Transmission electron microscopy analysis

The LAL muscles, dissected from P7 mice after drug treatment, were fixed on ice overnight with 2.5% glutaraldehyde and 4% paraformaldehyde (PFA) in about 1 cm × 1 cm size. Then the muscles were embedded using sandwich method (muscles were embedded between two sheets of glass slides) and treated as described in our recent study^73^. We prepared the muscle slice at 50 ∼ 70 nm thickness using LEICA EM UC7 and observed the images of NMJs with JEOL JEM-1230 TEM.

### TNFα treatment of LAL muscles

Purified TNFα proteins (10∼ 20 ng) in saline containing 0.1% BSA (v/v) were injected twice daily subcutaneously to the left LAL muscle of neonatal mice starting from P3. At P7 or P14, the whole left LAL muscles were isolated and subjected to TEM or immunostaining analysis. The left LAL muscles from mice injected with 0.1% BSA were used as control.

### Activity-dependent synaptic competition and time-lapse imaging

Motoneurons isolated from E13.5 rat spinal cord were transfected with ChR2-mCherry or YFP plasmids separately by in vitro electroporation using the Amaxa Nucleofector device, then mixed (1∼ 4 × 10^4^ cells/mL) and plated on differentiated primary muscle cells, and cultured for 24 h according to the protocol introduced previously^68,74,75^. The somas of ChR2-expressing motoneurons were stimulated with pulsed blue light (∼ 470 nm, 2 Hz, 5 ms per pulse, 10 pulses per trial with 60 s interval), followed immediately by time-lapse imaging. For dual-color imaging, excitation laser of 473 nm (Em: 490–560 nm) and 543 nm (Em: 570–620 nm) were employed. During stimulation, phase-contrast images and fluorescent images were recorded every 10 min with Z-series stack at 1.0 μm interval, using Olympus FV1000 confocal microscope with a ×40 water objective (Olympus). The morphology of motoneuron was reconstructed from images containing several *Z*-stacks (5 ∼ 10 stacks) and projected to two dimensions (2D) with maximum intensity.

### Immunohistochemistry, confocal microscopy image analysis, and statistics

LAL muscles were dissected after fixation with 4% PFA for 12 h at 4 °C and subjected to whole-mount staining with R-BTX and indicated antibodies following the procedure described previously^21,76^. The presynaptic nerve terminals were marked with antibodies against intermediate neurofilament (NF) and synaptic vesical protein synaptophysin or synapsin-1, and R-BTX to label postsynaptic AChR. Images were acquired on a NIKON A1R or TiE laser scanning confocal microscope with 1 µm interval in each stack, reconstructed to three dimensions (3D) containing several *Z*-stacks (40 ∼ 50 stacks) and projected to 2D with maximum intensity using Fiji software. Every single NMJ was observed with *Z*-stacks for better visualizing the number of innervated terminals.

To identify the subcellular localization of TNFα at NMJ, cross-sections of sternocleidomastoid muscles at 15 ∼ 20 µm thickness were subjected to staining with R-BTX and indicated antibodies. Images were acquired on a NIKON TiE laser scanning confocal microscope or LEICA TCS SP8 STED microscopy. In addition, whole sternocleidomastoid muscle was immunostained with R-BTX and antibodies against NF, βIII-Tubulin, and TNFR1. Images were acquired on a NIKON TiE laser scanning confocal microscope with 0.2 µm interval in each stack, reconstructed to 3D containing several *Z*-stacks (~20 stacks), and projected to 2D with maximum intensity using Fiji software. Post-acquisition images were processed with Fiji, Adobe Photoshop CC 2017, and Illustrator CS5 software. Data were quantitatively analyzed using Mann–Whitney test and are shown as means ± SEM from at least three experiments (*P* ≤ 0.05 was considered as significant difference).

### Activity-dependent expression of TNFα in vivo

C57BL/6 mice at P7 were anesthetized using pentobarbital sodium (40 mg/kg) during all treatments. For muscle transfection, 10 μg of plasmids driving exogenous expression of TNFα-pHluorin and mCherry were injected subcutaneously to the pectoralis superficial muscle, followed by electroporation consisting of eight square wave pulses with an amplitude of 60 v, a duration of 50 ms, and an interval of 1 s (ECM830; BTX). Then, the pups were placed back into the cage of mother mice. After 24 h, the pectoralis superficial muscle were dissected and placed in warmed ACSF buffer (124 mM NaCl, 2.5 mM KCl, 1.2 mM NaH_2_PO_4_, 24 mM NaHCO_3_, 5 mM HEPES, 12.5 mM Glucose, 2 mM MgSO_4_, 2 mM CaCl_2_). The medial pectoral nerve, which innervates the pectoralis superficial muscle, was peeled and stimulated with electrical current with an amplitude of 1 v (2 Hz, 5 ms per pulse, 10 pulses per train with 60 s interval) for 5 min in each trial. Meanwhile, live muscles were scanned with different lasers for the excitation of green (488 nm) and red (543 nm) signals, and fluorescence signals were viewed and collected using individual filter set (500–550 nm for green, 570–620 nm for red) under Nikon FN1 confocal microscope. For real-time observation, the multichannel signals were collected for durations of 5 min at an interval of 1 s.

## Supplementary information

Supplementary Information

Movie S1. Blue light-induced photo bleaching of mCherry signals in muscle cells

Movie S2. ChR2-gated muscle activation.

Movie S3. Photo bleaching of ChR2-mCherry after blue light application.

Movie S4. Muscle activation promotes TNFα secretion.

Movie S5. 3D Images for the expression of TNFR1 in NMJs

Movie S6. 3D images for the expression of TNFR1 in retracting axon terminals

Movie S7. Activity-dependent competition in motoneuron-muscle coculture system.

Movie S8. Time-lapse imaging for the dynamics of axons co-innervating same AChR patch.

Movie S9. Time-lapse imaging for the dynamics of axons co-innervating TNFα-deficient muscle cells.

Movie S10. Effect of DEVD on activity-dependent competition in motoneuron-muscle coculture system.

## Supplementary information

**Supplementary Information** accompanies the paper at (10.1038/s41421-020-0143-5).
