# Supplementary material for: Tumor necrosis factor alpha mediates neuromuscular synapse elimination
Source: Cell Discov. 2020 Mar 3;6:9. doi: 10.1038/s41421-020-0143-5 (PMC7051980; doi:10.1038/s41421-020-0143-5)
Supplement: Supplementary file 1 — Supplementary Information [file 41421_2020_143_MOESM1_ESM.docx]

Supplementary Information

Supplementary Figures


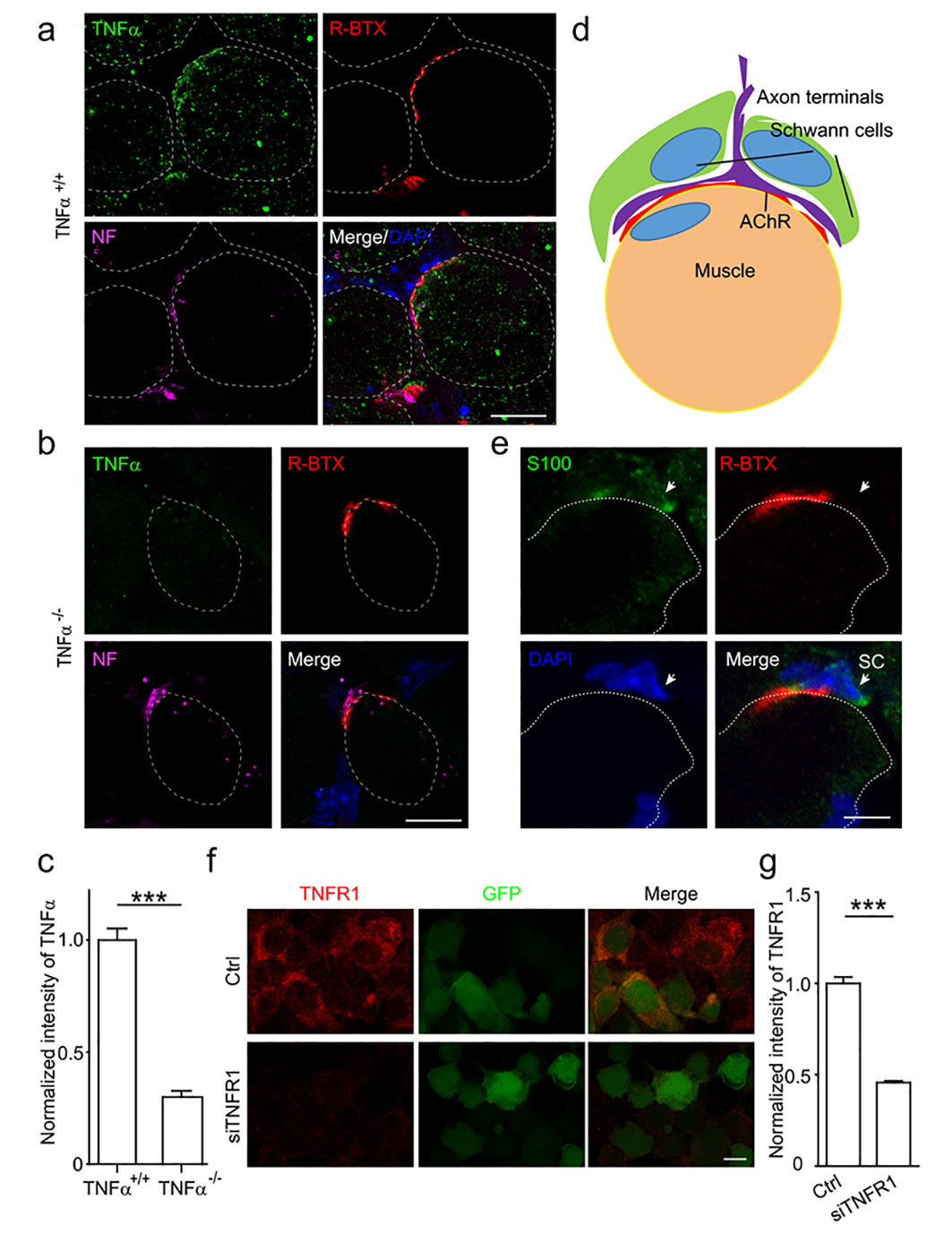


**Fig. S1 Spatial localization of TNFα and TNFR1 at the NMJ. (a, b)** Cross sections of *sternocleidomastoid* muscles at P6 from WT (a) or TNFα KO mice (b) were stained with R-BTX (red) and antibodies against TNFα (green) and NF (magenta). White dash lines delineate the membrane of myotubes. DAPI signals (blue) show the cell nuclei. Scale bars: 10 µm. **(c)** Quantification for normalized intensity of TNFα in muscles of WT and TNFα KO mice. Mann-Whitney test was used to determine significance. ****P* < 0.001. **(d)** Schematic representation of the NMJ composed of terminal SCs, presynaptic axon terminals, and post-synaptic membrane of muscles. **(e)** The terminal SCs (white arrow) labeled by S100 is near the NMJ marked by R-BTX. Scale bar: 5 µm. **(f)** HEK 293 cells were transfected with control or TNFR1 siRNA (siTNFR1) plasmids together with GFP and then stained with anti-TNFR1 antibody [(red)](#_ENREF_25). **(g)** Quantification for relative intensity of TNFR1. Data are shown as mean ± SEM (n =139 cells in control group, n = 102 cells in siTNFR1 group). Student’s *t* test was used to determine significance. ****P*<0.001.

­­
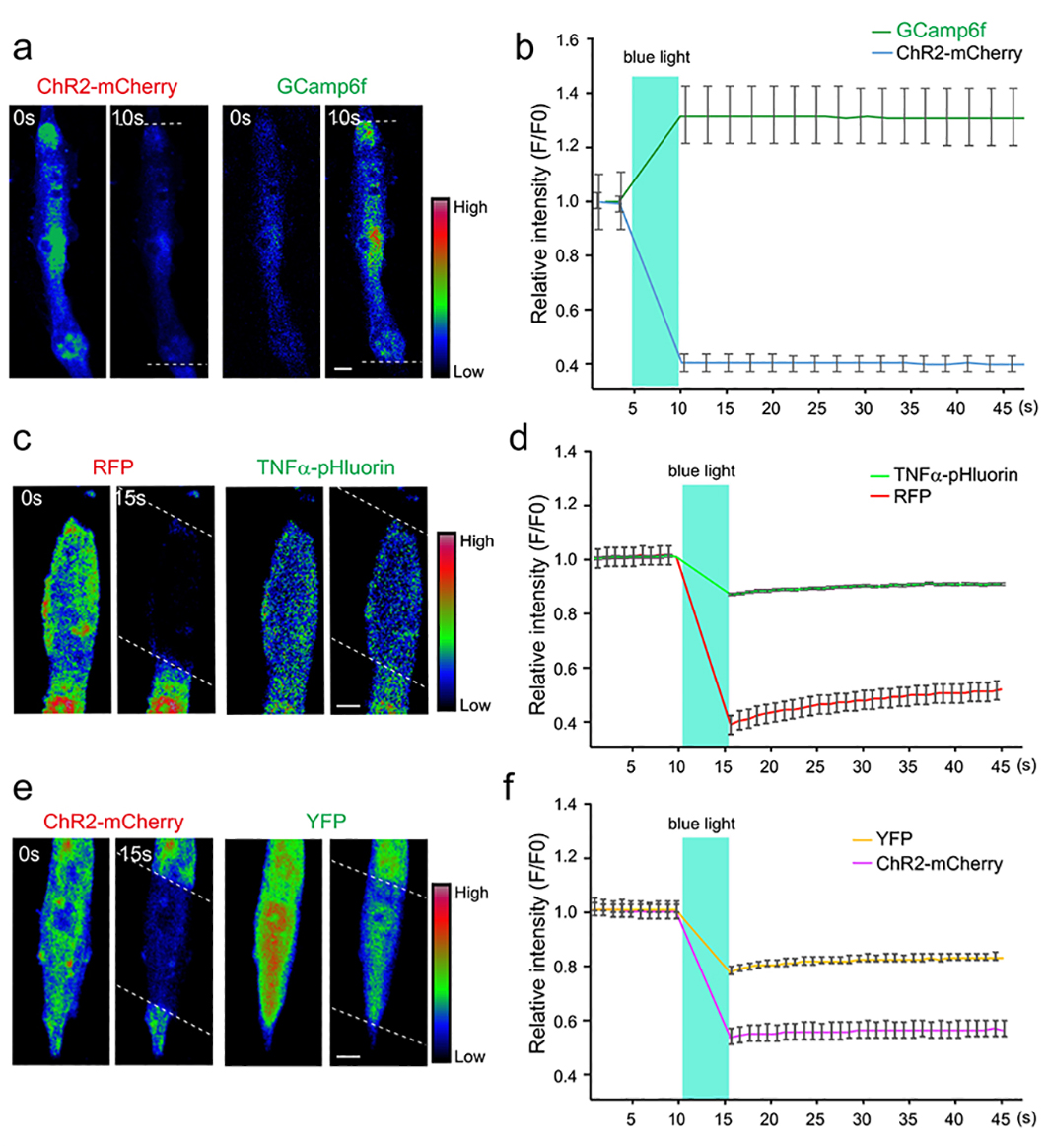


**Fig. S2 Muscle activation promotes TNFα secretion.** **(a, c, e)** C2C12 muscle cells co-expressing ChR2-mCherry and GCaMP6f (a), RFP and TNFα-pHluorin (c), or YFP and ChR2-mCherry (e) were stimulated with pulsed blue light (~470 nm). Scale bars: 20 µm (a) and 10 µm (c, e). **(b, d, f)** Mean intensity of 20 cells at each time point before and after light stimulation was quantified. Note the increase in calcium signals revealed by GCaMP6 and photo-bleach of other signals.


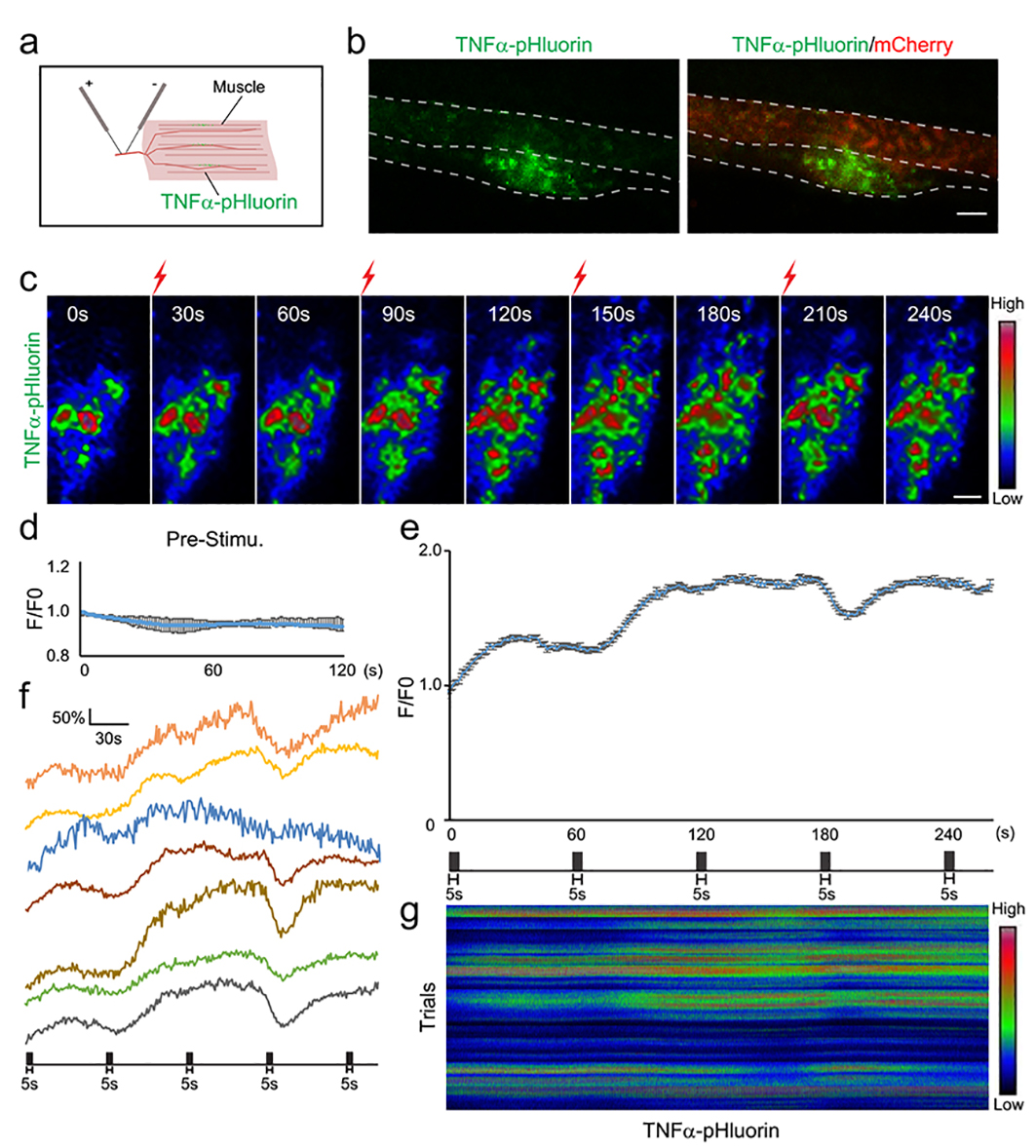


**Fig. S3 Muscle activation promotes TNFα secretion *in vivo.* (a)** Schematic representation of experimental setup. The *pectoralis superficial* muscles of P8 mice were transfected with TNFα-pHluorin plasmid using electroporation. After 24 hr, innervating nerves were subjected to electrical stimulation. **(b)** Expression of TNFα-pHluorin (green) and co-transfected mCherry [(red)](#_ENREF_25) in *pectoralis superficial* muscle cells. Scale bar: 5 μm. **(c)** Dynamic changes of TNFα-pHluorin signals in muscles upon sequential electrical stimulation of innervating nerves. Electrical stimuli were applied for 5 sec (red lightning) in an interval of 60 sec. Scale bar: 5 μm. **(d)** Fluorescence intensity of TNFα-pHluorin along the muscles prior to electrical stimulation. **(e)** Representative trials of TNFα-pHluorin responses in muscle cells during sequential electrical stimulation of innervating nerves. **(f)**  Mean response of 25 trials from 6 animals. **(g)** Heat map of individual trials.


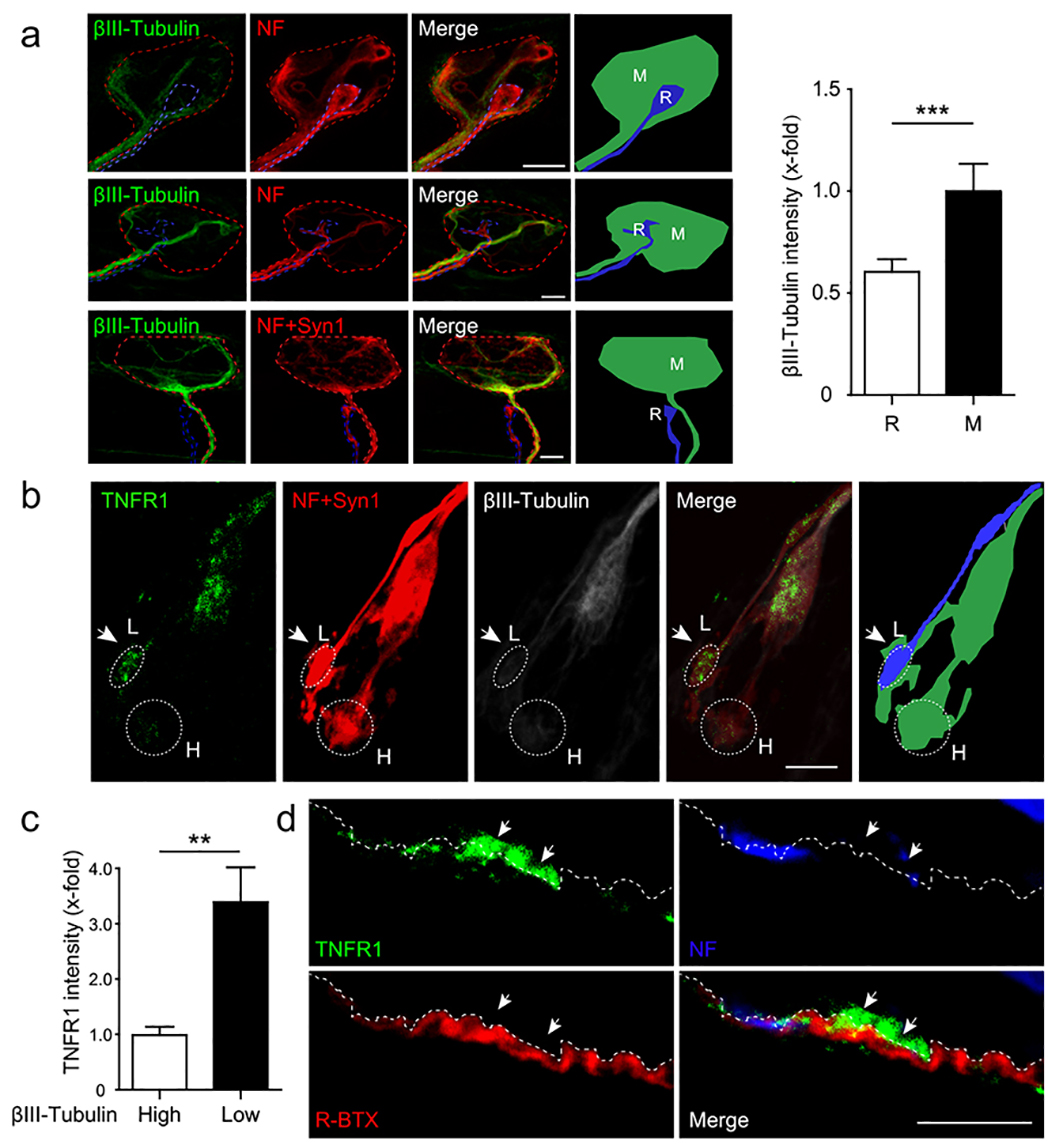


**Fig. S4 TNFR1 is mainly localized in retraction axon terminals at NMJs. (a)** The *sternocleidomastoid* muscles from P10 mice were stained with antibodies against βIII-Tubulin (green) and NF or NF plus Syn1 [(red)](#_ENREF_25). Note presumably retracting axons (outlined by blue lines) with condensed NF and low levels of βIII-Tubulin. Schematic diagrams reconfigure areas covered by axonal branches of presumably retracting (R) and maintained (M) terminals labelled by NF or NF and Syn1. Scale bar: 5 µm. Right panel shows quantification for relative intensity of βIII-Tubulin normalized with NF, with the value of maintained terminal set as 1. Data are shown as mean ± SEM of 25 NMJs from 4 mice. Mann-Whitney test was used to determine significance. ****P*<0.001. **(b)** The *Sternocleidomastoid* muscles from P10 mice were stained with TNFR1 (green), βIII-Tubulin [(red)](#_ENREF_25), and NF+Syn1 ([white](http://www.so.com/link?url=http%3A%2F%2Fdict.youdao.com%2Fsearch%3Fq%3Dmagenta%26keyfrom%3Dhao360&q=magenta&ts=1510041928&t=9436af0290e96e2cc493ed2a99e5763)). Scale bar: 5 µm. Areas covered by presumably retracting (R) and maintained (M) terminals were re-configurated in the schematic diagram. **(c)** Quantification for normalized intensity of TNFR1 relative to NF/Syn1 in terminals (white dash circle) of axons with high and low βIII-Tubulin levels in axonal trunks. Data are shown as mean ± SEM of 15 NMJs from 4 mice. Mann-Whitney test was used to determine significance. ***P*<0.01. **(d)** The *Sternocleidomastoid* muscles from P10 mice were stained with R-BTX [(red)](#_ENREF_25) and antibodies against TNFR1 (green) and NF (blue). Note the localization of TNFR1 in the pre-synaptic nerve terminal (white arrows). Scale bar: 5 µm.


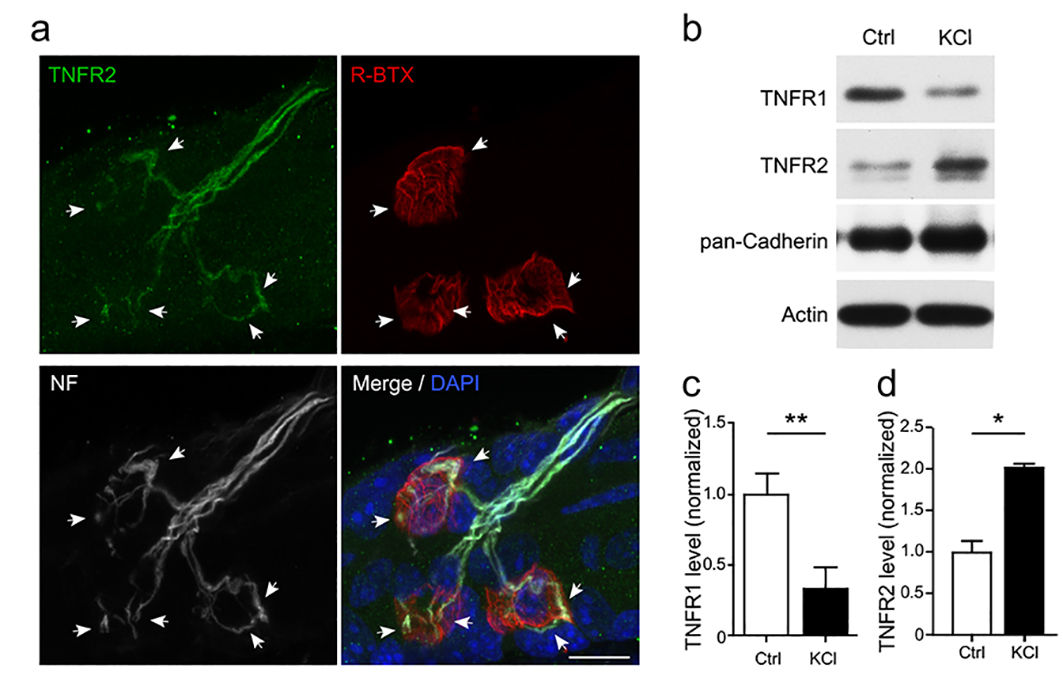


**Fig. S5 Expression of TNF receptors in motor neurons. (a**) The *Sternocleidomastoid* muscles from P10 mice were stained with R-BTX (red) and antibodies against TNFR2 (green) and NF (white). Note the expression of TNFR2 (white arrows) in axonal branches and terminals opposed to AChR patches. DAPI signals (blue) show the cell nuclei. Scale bar: 20 µm. **(b**) Cultured mouse motoneurons were treated with 50 mM KCl for 30 min then membrane fractions were subjected to IB with indicated antibodies. **(c, d**) Normalized level of membrane TNFR1 (c) or TNFR2 (d) relative to pan-Cadherin was presented as mean ± SEM from 3 experiments. Mann-Whitney test was used to determine significance. **P<*0.05, ***P*< 0.01*.*


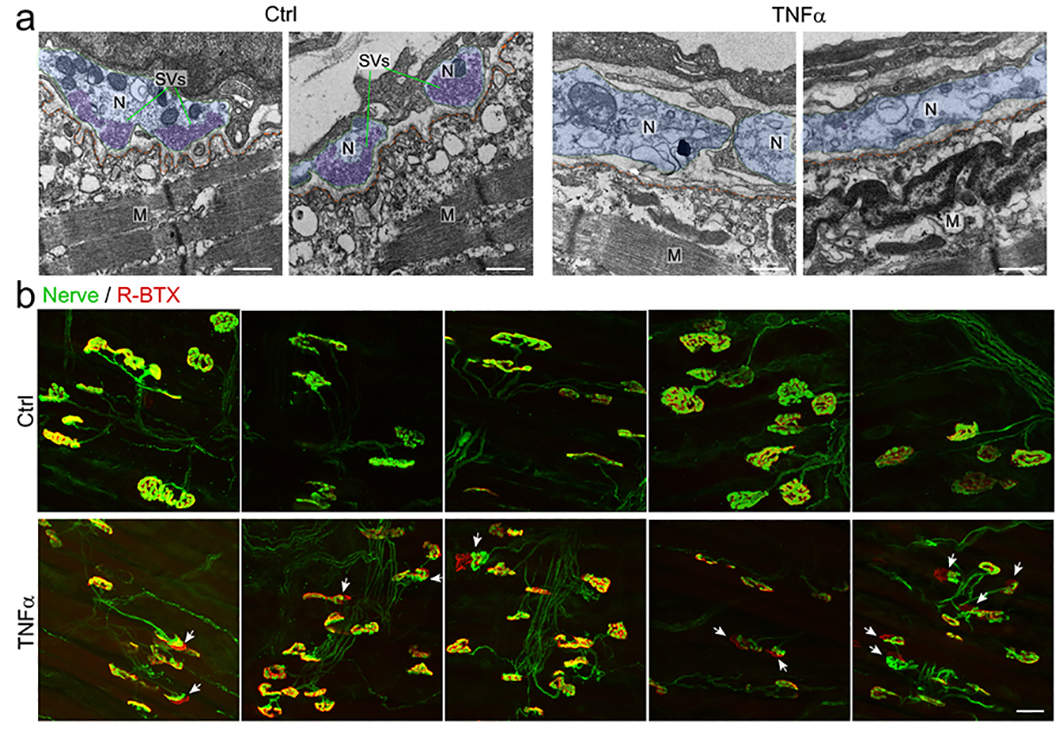


**Fig. S6 TNFα treatment results in abnormal NMJs. (a)** EM images of NMJs after BSA (Ctrl) or TNFα treatment at P7. N, nerve; M, muscle; SVs, synaptic vesicles. Scale bar: 0.5 µm. **(b)** LAL muscles of mice at P14 treated with BSA (Ctrl) or TNFα were stained with R-BTX (red) and antibodies against NF plus SYP (Nerve, green). Note the disassociation of axon terminals with AChR patches in TNFα-treated group (white arrows). Scale bar: 20 µm.


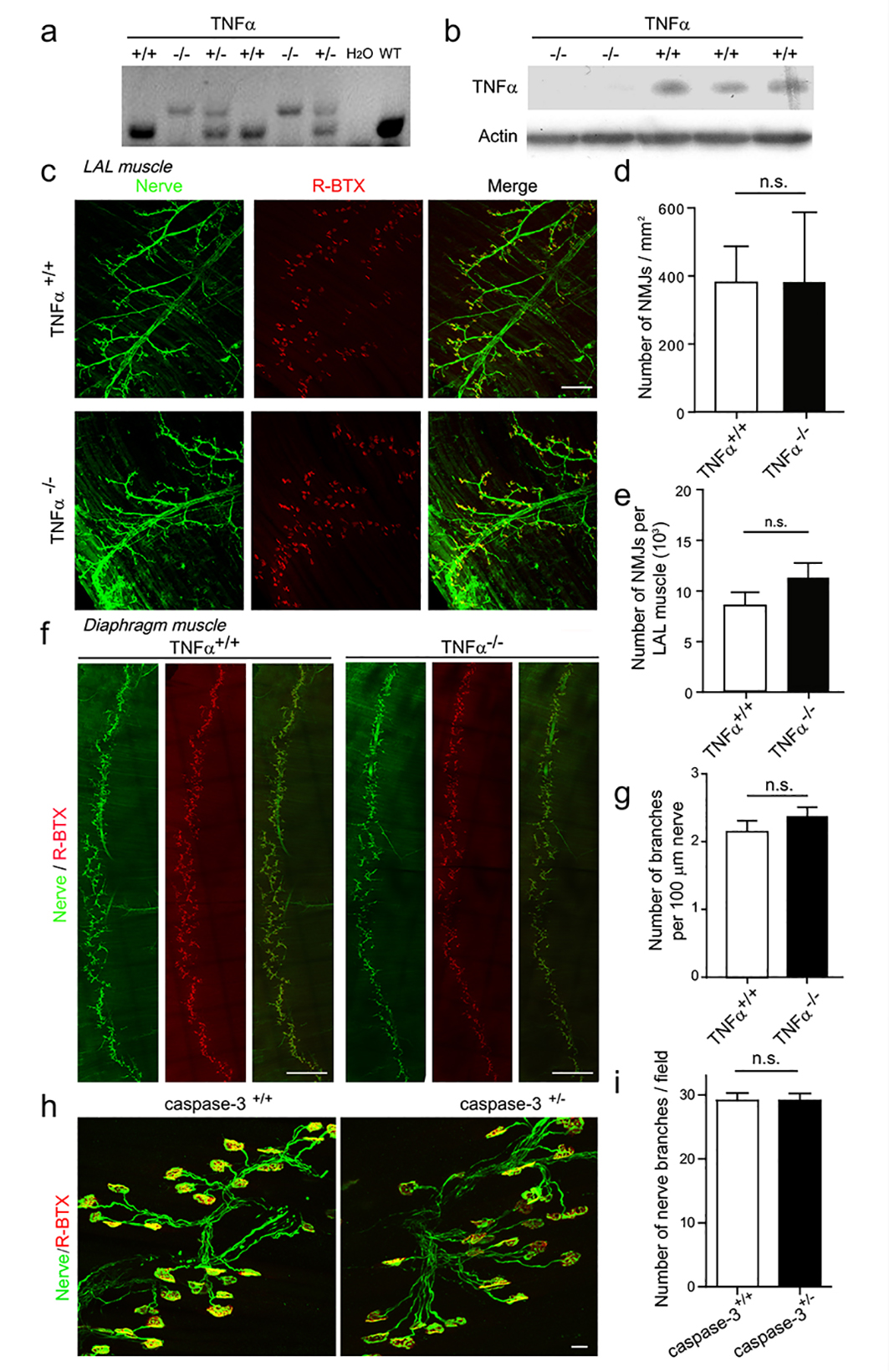


**Fig. S7 NMJ formation and axon patterning in *TNFα* and *caspase-3* mutant mice. (a, b)** Genotyping of TNFα knockout mice using PCR (a) or immunoblotting (b)*.* **(c)** LAL muscles from WT (*TNFα^+/+^*) or *TNFα* knockout (*TNFα^-/-^*) mice at P0 were stained with R-BTX (red), and axonal markers NF plus SYP (Nerve, green). Scale bars: 20 µm. **(d, e)** Quantification for the numbers of NMJs per mm^2^ (d) and total NMJ numbers (e) in each LAL muscles from indicated genotypes. Data are shown as mean ± SEM from 4 mice in each group. Mann-Whitney test was used to determine significance. n.s., no significant difference. **(f)** *Diaphragm* muscles from P0 mice of indicated genotypes were stained with R-BTX (red), and axonal markers (SYP, green). Scale bars: 50 µm. **(g)** Quantification of the number of axonal branches emanating from 100 µm nerve trunk in the diaphragm of P0 mice. Data are shown as mean ± SEM from 4 mice in each group. Mann-Whitney test was used to determine significance. n.s., no significant difference. **(h)** LAL muscles from wildtype or *caspase-3^+/-^* mice at P9 were stained with R-BTX [(red)](#_ENREF_25) and antibodies against NF and Syn1 (Nerve, green). Scale bar: 20 µm. **(i)** Quantification of the number of axonal branches in the LAL of P9 mice. Data are shown as mean ± SEM from 4 mice in each group. Mann-Whitney test was used to determine significance. n.s., no significant difference.


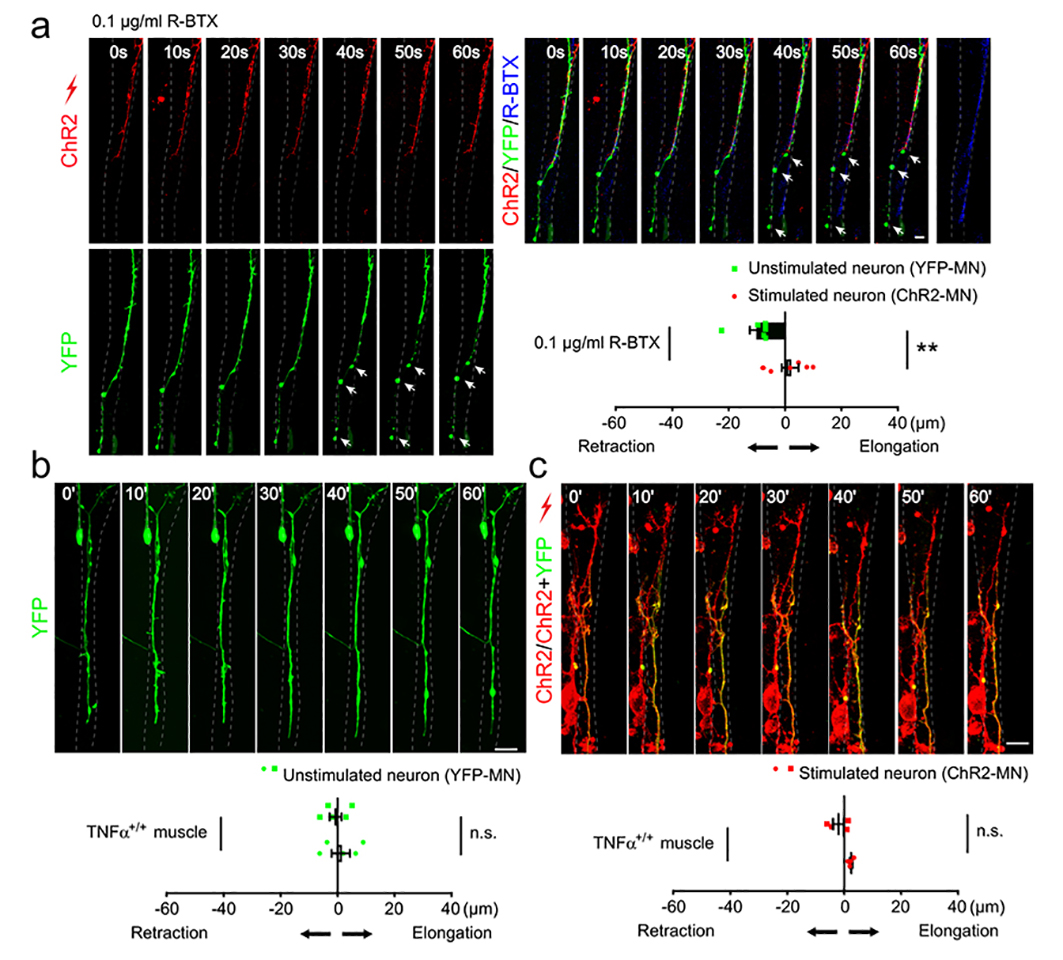


**Fig. S8 Triplet set-up for optogenetic manipulation of cultured motoneurons on muscles. (a)** Triplet cultures composed of muscle cells, ChR2- and YFP-expressing motoneurons (ChR2-MN and YFP-MN) were incubated with low concentration of Alex-647-labeled R-BTX (0.1 µg/ml, blue) and subjected to blue light stimulation on the soma of ChR2-MN. Time-lapse images were taken every 10 min after light stimulation. Note the retraction bulbs (arrows) of YFP (green) terminal after light stimulation of ChR2 (red) neuron during the observation period. Scale bar: 10 µm. The diagram shows quantification for the distance of axonal retraction or elongation in 60 min after blue light stimulation of ChR2-MN in the triplets. Data are shown as mean ± SEM of values from 6 samples. Mann-Whitney test was used to determine significance. ***P* < 0.01. **(b)** Competition analysis in situations with two YFP-MN co-innervating one muscle cell. Time-lapse images were taken every 10 min without light stimulation. Scale bar: 10 µm. Data are shown as mean ± SEM of values from 5 samples. Mann-Whitney test was used to determine significance. n.s., no significant difference. **(c)** Competition analysis in situations with two ChR2-MN co-innervating one muscle cell. Time-lapse images were taken every 10 min after light stimulation on the somas of both ChR2-MN. Scale bar: 10 µm. Data are shown as mean ± SEM of values from 4 samples. Mann-Whitney test was used to determine significance. n.s., no significant difference.

**Supplementary Movies**

**Movie S1. Blue light-induced photo bleaching of mCherry signals in muscle cells.** C2C12 myotubes co-transfected with ChR2-mCherry and GCaMP6f were stimulated with ~470 nm laser to gate the ChR2 channel to activate muscle cells, followed by time-lapse imaging of mCherry signals (543 nm excitation, 570-620 nm emission). Note the decrease in mCherry signals caused by photo bleaching.

**Movie S2. ChR2-gated muscle activation.** C2C12 myotubes co-transfected with ChR2-mCherry and GCaMP6f were stimulated with ~470 nm laser to gate the ChR2 channel to activate muscle cells, followed by time-lapse imaging of calcium signals revealed by GCaMP6 (488 nm excitation, 500-550 nm emission).

**Movie S3. Photo bleaching of ChR2-mCherry after blue light application.** C2C12 myotubes co-transfected with TNFα-pHluorin and ChR2-mCherry were stimulated with ~470 nm laser to gate the ChR2 channel to activate muscle cells, followed by time-lapse imaging of mCherry signals (543 nm excitation, 570-620 nm emission). Note the decrease in mCherry signals caused by photo bleaching.

**Movie S4.** **Muscle activation promotes TNFα secretion.** C2C12 myotubes co-transfected with TNFα-pHluorin and ChR2-mCherry were stimulated with ~470 nm laser to gate the ChR2 channel to activate muscle cells, followed by time-lapse imaging of pHluorin signals (488 nm excitation, 500-550 nm emission). Note the increase in pHluorin signals upon blue light stimulation.

**Movie S5. 3D Images for the expression of TNFR1 in NMJs.** Note the higher expression of TNFR1 (green) in the tip of retracting axon terminal but not maintained terminal, which co-innervated the same AChR patch (blue). Axons were labeled by NF (red).

**Movie S6. 3D images for the expression of TNFR1 in retracting axon terminals.** Note the higher expression of TNFR1 (green) in the tip of retracting axon terminal but not maintained terminal. Axons were labeled by NF and Syn1 (red).

**Movie S7. Activity-dependent competition in motoneuron-muscle coculture system.** Triplet cultures composed of ChR2-mCherry-expressing motoneuron (ChR2-MN, red), YFP-expressing motoneuron (YFP-MN, green), and muscle cells were subjected to blue light stimulation. Time-lapse images were taken every 10 min after light stimulation. For dual-color imaging, excitation laser of 473 nm (Em: 490-560 nm) and 543 nm (Em: 570-620 nm) were used. Note the retraction of YFP terminal after light stimulation of ChR2 neuron during the observation period.

**Movie S8. Time-lapse imaging for the dynamics of axons co-innervating same AChR patch.** Triplet cultures composed of ChR2-MN, YFP-MN, and muscle cells were incubated with low concentration of Alex-647-labeled R-BTX (0.1 µg/ml, blue) and subjected to blue light stimulation on the soma of ChR2-MN, followed by time-lapse imaging of three signals. Note the retraction bulbs of YFP terminal after light stimulation of ChR2 neuron during the observation period.

**Movie S9. Time-lapse imaging for the dynamics of axons co-innervating TNFα-deficient muscle cells.** Triplet cultures composed of ChR2-MN (red), YFP-MN (green), and TNFα deficient muscle cells were subjected to blue light stimulation. Time-lapse images were taken every 10 min after light stimulation. Note the elongation of both terminals contacted with *TNFα^-/-^* muscle cells during the observation period.

**Movie S10. Effect of DEVD on activity-dependent competition in motoneuron-muscle coculture system.**
